# A Robust and Versatile QM/MM Interface for Molecular Dynamics in GROMOS

**DOI:** 10.1002/jcc.70053

**Published:** 2025-02-07

**Authors:** Peter Poliak, Patrick Bleiziffer, Felix Pultar, Sereina Riniker, Chris Oostenbrink

**Affiliations:** ^1^ Institute of Molecular Modeling and Simulation, Department of Material Sciences and Process Engineering University of Natural Resources and Life Sciences Vienna Vienna Austria; ^2^ Institute of Physical Chemistry and Chemical Physics, Faculty of Chemical and Food Technology Slovak University of Technology Bratislava Slovakia; ^3^ Department of Chemistry and Applied Biosciences ETH Zürich Zürich Switzerland; ^4^ maXerial AG Vaduz Liechtenstein; ^5^ Christian Doppler Laboratory for Molecular Informatics in the Biosciences University of Natural Resources and Life Sciences Vienna Austria

**Keywords:** density functional theory, embedding schemes, link‐atom scheme, semiempirical methods, solvated amino acids, SPC water

## Abstract

The integration of quantum mechanics and molecular mechanics (QM/MM) within molecular dynamics simulations is crucial to accurately model complex biochemical systems. Here, we present an enhanced implementation of the QM/MM interface in the GROMOS simulation package, introducing significant improvements in functionality and user control. We present new features, including the link atom scheme, which allows the modeling of QM regions as a part of bigger molecules. Benchmark tests on various systems, including QM water in water, amino acids in water, and tripeptides validate the reliability of the new functionalities. Performance evaluations demonstrate that the updated implementation is efficient, with the primary computational burden attributed to the QM program rather than the QM/MM interface or the MD program itself. The improved QM/MM interface enables more advanced investigations into biomolecular reactivity, enzyme catalysis, and other phenomena requiring detailed quantum mechanical treatment within classical simulations. This work represents a significant advancement in the capabilities of GROMOS, providing enhanced tools to explore complex molecular systems.

## Graphical abstract

We present an enhanced QM/MM interface in the GROMOS package, featuring improved functionality, including a link atom scheme for QM regions within larger molecules. Benchmark tests of water and amino acids in water, and dihedral potential energy scans in vacuo, validate the reliability and efficiency of the new implementation, enabling advanced studies in biomolecular reactivity and enzyme catalysis through integrated quantum‐classical simulations.

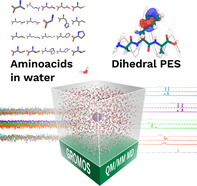

**Abbreviations**
DFT: density functional theory
EE: electrostatic embedding
LJ: Lennard‐Jones
MECC: mechanical embedding with constant charges
MEDC: mechanical embedding with dynamic charges

## 1 Introduction

Molecular dynamics (MD) simulations play a crucial role in many biochemical discoveries, providing insights into the structural dynamics of biomolecules that would be otherwise unattainable. For nearly 50 years, classical Newtonian molecular mechanics (MM) using force fields has been fundamental to the study of proteins [1]. The continuous growth in computational power has allowed for significant improvements in the accuracy of potential energy functions through better parameterization, the inclusion of previously neglected effects, and the application of quantum mechanics (QM) [2]. Recently, advancements have further incorporated machine learned potentials (MLPs) into these models [3, 4]. QM/MM methods were already introduced just a year before the first protein MD simulation [5], but their practical use became feasible only in recent decades with the increasing performance of computers [6, 7, 8, 9, 10, 11]. Nowadays, QM/MM features are standard in many molecular simulation programs, including GROMOS [12].

The GROMOS program package [13, 14, 15] is a versatile molecular simulation software package, that offers many well‐established and novel functionalities for performing MD simulations. Besides using common MM force fields, GROMOS allows simulations where part of the molecular system is described at the QM level. This QM/MM approach, first introduced by Warshel and Levitt in 1976, combines these two physical approaches to enhance the physico‐chemical description of molecular systems. QM/MM is particularly useful for studying systems that change their electron configuration during processes such as chemical reactions and excited state dynamics, or when reasonable MM parameters are unavailable. The inclusion of QM/MM features, now standard in many molecular simulation programs, exemplifies the advanced capabilities of GROMOS.

The GROMOS program specializes in force‐field molecular simulations. Undoubtedly, writing yet another QM implementation with a lot of available ready‐to‐use QM programs is unnecessary. Instead, GROMOS offloads QM calculations to the external quantum‐mechanical program. The first implementation contained interfaces to two QM packages, MNDO and Turbomole [12]. The interface written in C++ is responsible for generating corresponding input files for the external QM program, calling the program, and reading and parsing its output files. In comparison to a directly incorporated source code, or library linking, this approach gives users more control over methods and keywords specifications. It is also less dependent on the QM programs development, since newly implemented methods can be used directly. For high‐performance with computationally efficient methods in xtb [16], we have linked the program directly to the corresponding C API libraries. Similarly, the Schnetpack [17] interface links directly to its Python library with the use of pybind11 library [18].

In this paper, we present extended QM/MM functionalities of the GROMOS software and new interfaces to additional QM programs. We reworked the code to give the user more control over the method selection, such as embedding schemes, inclusion of van der Waals interactions, and we also introduced the possibility to bond QM and MM regions covalently using a link‐atom scheme. By taking advantages of object‐oriented programming in C++, we restructured the code such that any prospective new features are now easier to implement. Adding a new specialized embedding scheme, an interface to another QM program, or an alternative link‐atom scheme can be easily achieved by writing a specialized child class.

We have implemented interfaces to six additional QM software packages and one neural network toolbox—MOPAC [19], DFTB+ [20], xtb [16], Gaussian 16 [21], ORCA [22], and Schnetpack [17]. We have also reworked the already implemented MNDO [23] and Turbomole [24] interfaces [12] to comply with the new framework. The supported and tested versions of the QM software packages are specified in Table S1. New features now allow users to:

- Choose between an atomic or charge‐group based cutoff scheme to include MM atoms within electrostatic embedding (EE).
- Determine whether a Lennard‐Jones potential should be applied between QM atoms. This feature provides experienced users with full control over dispersion interactions within the QM region, addressing the partial lack of dispersion in lower‐level QM methods. Default force‐field parameters are likely unsuitable and should be adjusted to meet the user's specific requirements.
- Decide if bond length constraints should be applied between the QM atoms.
- Set whether the charges on QM atoms are updated from the QM calculation or are kept constant.
- Scale the MM charges based on distance to the closest QM atom as
(1) $$q_{\mathrm{MM}}^{\prime }=q_{\mathrm{MM}}\frac{2}{\pi }\arctan\left(\mathrm{sd}\right)$$
  where *s* is a user‐specified parameter activated by setting *s* > 0 and *d* is the distance to the closest QM atom. MM charges located near QM atoms can induce artifacts due to overpolarization [25], and this feature provides a means to mitigate it. However, as an experimental feature, it should be used with caution.

The QM/MM approach is supposed to improve efficiency of the calculation. Ideally, the results should approximate the full QM level calculation, but at much lower computational cost. Obviously, this is a naïve expectation, however, in certain cases a well set up QM/MM can yield outstanding results [11]. As a prerequisite, the chemically important part should be entirely in the QM zone and the interactions with the rest should be well parameterizable [26, 27]. Selection of the QM zone has its pitfalls, is a broad topic, and is certainly beyond the scope of this article. Instead, the aim of this work is to present the improved implementation of the QM/MM engine in GROMOS and validate the implementation and its robustness on simple physico‐chemical systems.

We performed extensive tests to validate the presented implementation. All new QM program interfaces passed basic sanity checks and were successfully used to run short simulations. We tested various combinations of QM programs and embedding schemes. In this work, we report only selected results to demonstrate the correct functioning of the program.

In this work, we present results from QM/MM simulations of three types of systems:

1. A single QM water in a periodic box of SPC water
2. A single amino acid residue in a periodic box of SPC water
3. Five different alanine‐X‐alanine tripeptides in vacuo, where X is aspartic acid (ADA), phenylalanine (AFA), histamine (AHA), threonine (ATA), and valine (AVA)

The first test system allows to evaluate consistency between the QM and MM potential energy surfaces. In an ideal case, one would expect the interactions between the molecules on different levels of theory to be equivalent. Unfortunately, this does not hold true, and our goal was to monitor the extend of the discrepancy during the simulation.

The second system extends the previous one to more biologically relevant systems, since to simulate proteins in water, consistent interactions of QM amino acid molecules with SPC, or other water models is necessary.

The third test was selected to represent typical properties of aliphatic, aromatic, polar and non‐polar residues. The QM regions consist of the sidechain of the central amino acids, starting from C_β_. Here we performed a dihedral scan around the bond crossing the QM–MM boundary to validate the link atom scheme and to compare several full QM and full MM methods to the QM/MM approach.

The presented program update opens possibilities to perform advanced simulations of biomolecules, elucidate the enzyme activity, and other phenomena, where a detailed quantum description is crucial. Furthermore, it opens possibilities to perform free‐energy calculations, which should be preferred over energy‐minimization based reaction simulations in enzymatic studies [28, 29]. The GROMOS software package is freely available at www.gromos.net and https://github.com/biomos [15].

## 2 Theoretical Basis

In the QM/MM calculation, we split the system into at least two subsystems:

1. The QM zone, where the atoms are described by quantum mechanics.
2. The rest is the MM zone driven by classical mechanics.

Because of the significant difference between the masses of electrons and nuclei, the nuclear interactions are usually treated as classical coulombic within the Born‐Oppenheimer approximation, and only the electrons are treated quantum‐mechanically. Nevertheless, this significantly increases the computational costs due to the vast number of electrons and the need to solve the Schrödinger equation for the system iteratively in a self‐consistent manner. In GROMOS, the QM/MM energy is evaluated according to the equations already presented in the work of Meier et al. [12].

The new implementation allows the user to choose between three common QM/MM embedding schemes:

- Mechanical embedding (ME)—QM/MM interaction is calculated purely at the MM level. Charges of the QM atoms can be constant and specified by the force field using ME with constant charges (MECC), or we can allow to update the charges from the QM calculation every step in ME with dynamic charges (MEDC). Although temptingly elegant and computationally cheap, the dynamic charges induce additional forces on the QM atoms from the MM charge distribution [9]. The analytical form of these forces is typically not available, and we find this issue is often sparsely addressed in the literature and program documentation. As discussed in detail in the Supporting Information, constant charges are strongly recommended.
- Electrostatic embedding (EE)—QM/MM interaction is calculated at the QM level by providing all MM atoms within the specified cutoff radius from the QM atoms to the QM program of choice as point charges. This scheme implicitly includes electronic polarization of the QM zone by the MM atoms, but not vice versa.
- Polarizable embedding (PE)—this scheme is like EE, except the MM atoms are treated within the polarizable charge‐on‐spring formalism [30]. The nucleus charge is bound to the atom position while the charge‐on‐spring, is free to move following Hooke's law and a preset force constant. The charges‐on‐springs' positions are optimized iteratively every step to achieve self‐consistency together with the QM zone. Because several QM calculations per step are needed, this scheme is computationally very demanding. On the other hand, it covers mutual polarization of the MM and QM atoms. GROMOS supports charge‐on‐spring polarizable force fields.

Beside the aforementioned schemes, GROMOS also allows a specialized buffered embedding, called BuRNN [3], where an additional region between QM and MM is defined, in which mutual interactions are described by MM, but the interactions between the inner region and the buffer are described on the QM level. This approach is akin to the PE, while it takes advantage of and allows for the use of MLPs trained on QM data. It effectively allows mutual polarization between the inner and buffer region, while the transition between the inner and the outer region is more gradual.

### 2.1 Link Atom Scheme

All MM interactions that may be defined in the topology for atoms in the QM zone are removed from the MM calculation. Exceptions are the possibility to activate Lennard‐Jones interactions within the QM region (as a dispersion correction), or the possibility to maintain bond‐length constraints according to the optimal (MM) bond lengths. In case covalent bonds between the zones are specified, a QM calculation cannot be performed directly. A MM atom bonded to a QM atom should not be included in any form in the QM calculation, as this would essentially lead to double counting of the interactions. Moreover, the valence of the linked atom is incomplete due to missing bonded atoms, which would result in an ill‐defined system. To solve this, we split the bond for separate QM and MM calculation by using a link‐atom approach and keep some of the classical bonded terms which go over the QM/MM boundary. The nonbonded terms may be also treated in a special way. Several methods have been developed to redistribute the charges close to the link to avoid any close contact between the point charges and the QM atoms and their general overview is presented in reference [31]. In this work, we do not redistribute the charges of the linked MM atom. In our test system, the link atoms possess no charge within the used force field, rendering this aspect less critical. Implementation of charge redistribution schemes are a subject of further program development.

**FIGURE 1:**
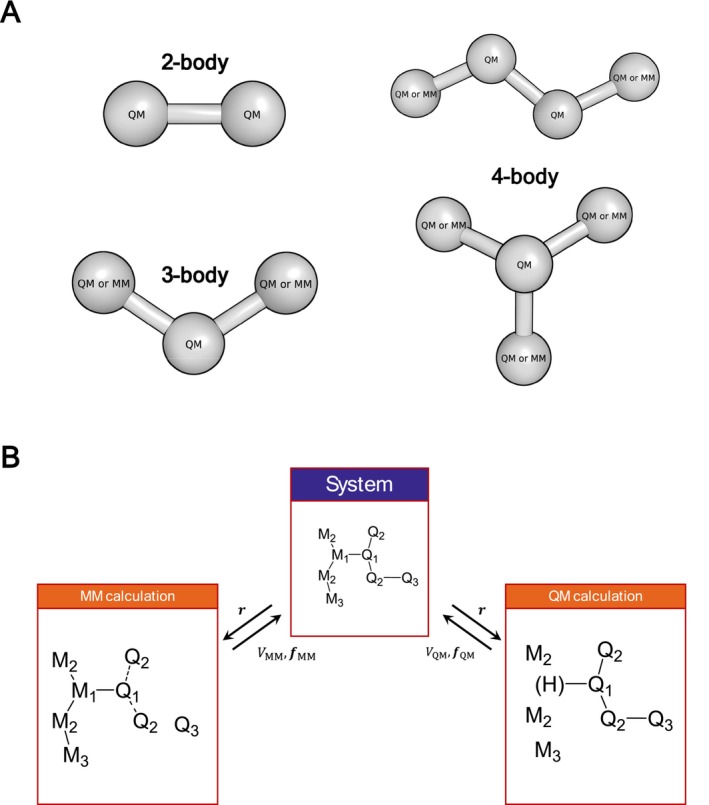
Interaction terms separation in the link atom scheme algorithm. (A) Representation of force field bonded terms, that are deactivated in the QM/MM calculation. (B) Splitting of the system within the link atom scheme. The indices of the atoms in the scheme represent the topological distance from the link. The solid line represents treatment of the given bond at the given level. The dashed line in the MM calculation represents dihedral terms only. The Q_*i*_–Q_*j*_ bonds are treated solely at the QM level, that is no classical bonds are present between them in the MM calculation. In mechanical embedding, the M_*i*_–Q_*j*_ coulombic interactions are treated only by the MM calculation, and in electrostatic embedding only by the QM calculation. The capping hydrogen atom, in brackets, is visible only to the QM atoms.

As shown in Figure 1A, only bonded force‐field terms, that ought to be well described by the QM calculation, are deactivated.

In case of two‐body terms representing the bond stretching, we deactivate them only if two QM atoms are involved. The QM–MM bonds must be defined in the force field. Bending angle three‐body terms are deactivated, only if the central atom is a QM atom. Bond torsion four‐body terms are deactivated, if the two central atoms are QM atoms. Improper dihedral four‐body terms are deactivated in case the central atom is a QM atom. In all these cases, the MM atoms will be replaced by capping hydrogen atoms, and proper conformations will be maintained by the QM calculation. The user should define the QM/MM boundary such that the links are applied on single covalent bonds only.

Figure 1B depicts the link‐atom scheme algorithm and the way the system is split for separate calculations. The classical dihedral terms are kept active up to the second QM atom, Q_2_. The MM atoms are represented only by point charges in the QM calculation. The atom M_1_ taking part in the QM–MM bond is completely hidden and the capping hydrogen atom is placed at a constant distance from the corresponding QM atom along the link,

(2) $$\begin{array}{c} r_{L}=r_{\mathrm{QM}}+d_{L-\mathrm{QM}}\frac{r_{\mathrm{MM}}-r_{\mathrm{QM}}}{\left|r_{\mathrm{MM}}-r_{\mathrm{QM}}\right|}\; \end{array}$$

where $r_{\mathrm{QM}}$, $r_{\mathrm{MM}}$ and $r_{L}$ are coordinates of the QM, MM and capping atom respectively, and $d_{L-\mathrm{QM}}$ is the constant distance between the capping atom and the QM atom, specified by the user. The capping atoms inherently contribute to the potential energy and interact with other atoms in the system. To conserve the force and the energy of the system and to eliminate the redundant degrees of freedom, the force acting on the capping atom is distributed to the QM and MM atom by applying the chain rule:

(3) $$\begin{array}{c} V\left(r_{L}\right)=V\left(r_{L}\left(r_{\mathrm{QM}},r_{\mathrm{MM}}\right)\right)\; \end{array}$$

(4) $$\begin{array}{c} f_{\mathrm{MM}}^{L}=\frac{-\partial V\left(r_{L}\right)}{\partial r_{\mathrm{MM}}}=\frac{-\partial V\left(r_{L}\right)}{\partial r_{L}}\frac{\partial r_{L}}{\partial r_{\mathrm{MM}}}=f_{L}\frac{\partial r_{L}}{\partial r_{\mathrm{MM}}}\; \end{array}$$

(5) $$\begin{array}{c} f_{\mathrm{QM}}^{L}=f_{L}\frac{\partial r_{L}}{\partial r_{\mathrm{QM}}} \end{array}$$

where $f_{L}$ is the total force on the capping atom, calculated on the QM level, $f_{\mathrm{MM}}^{L}$ and $f_{\mathrm{QM}}^{L}$ are its components distributed to the MM atom and the QM atom of the link, respectively, and $\frac{\partial r_{L}}{\partial r_{\mathrm{MM}}}$, $\frac{\partial r_{L}}{\partial r_{\mathrm{QM}}}$ are Jacobian matrices in the form.

(6) $$\begin{array}{c} {\left.J_{\mathrm{MM}}^{\alpha \beta }=\frac{\partial r_{L}^{\alpha }}{\partial r_{\mathrm{MM}}^{\beta }}=\frac{d_{L-\mathrm{QM}}}{d_{\mathrm{MM}-\mathrm{QM}}}\delta_{\alpha \beta }-\frac{d_{L-\mathrm{QM}}}{d_{\mathrm{MM}-\mathrm{QM}}}{\hat{r}}_{\mathrm{MM}-\mathrm{QM}}^{\alpha }{\hat{r}}_{\mathrm{MM}-\mathrm{QM}}^{\beta }\right|}_{\alpha ,\beta \;\epsilon \;\left\{x,y,z\right\}}\; \end{array}$$

(7) $$\begin{array}{c} {\left.J_{\mathrm{QM}}^{\alpha \beta }=\frac{\partial r_{L}^{\alpha }}{\partial r_{\mathrm{MM}}^{\beta }}=\left(1-\frac{d_{L-\mathrm{QM}}}{d_{\mathrm{MM}-\mathrm{QM}}}\right)\delta_{\alpha \beta }+\frac{d_{L-\mathrm{QM}}}{d_{\mathrm{MM}-\mathrm{QM}}}{\hat{r}}_{\mathrm{MM}-\mathrm{QM}}^{\alpha }{\hat{r}}_{\mathrm{MM}-\mathrm{QM}}^{\beta }\right|}_{\alpha ,\beta \;\epsilon \;\left\{x,y,z\right\}}\; \end{array}$$

where $d_{\mathrm{MM}-\mathrm{QM}}$ is the distance between the MM and QM atom and ${\hat{r}}_{\mathrm{MM}-\mathrm{QM}}^{\alpha }$, ${\hat{r}}_{\mathrm{MM}-\mathrm{QM}}^{\beta }$ are respective components of the unit vector pointing from the QM atom to the MM atom. Equations (4) and (5) can be thus rewritten as

(8) $$\begin{array}{c} f_{\mathrm{MM}}^{L}=f_{L}\frac{d_{L-\mathrm{QM}}}{d_{\mathrm{MM}-\mathrm{QM}}}-b\; \end{array}$$

(9) $$\begin{array}{c} f_{\mathrm{QM}}^{L}=f_{L}\left(1-\frac{d_{L-\mathrm{QM}}}{d_{\mathrm{MM}-\mathrm{QM}}}\right)+b\; \end{array}$$

(10) $$\begin{array}{c} b=\frac{d_{L-\mathrm{QM}}}{{d_{\mathrm{MM}-\mathrm{QM}}}^{3}}\left(f_{L}\times r_{\mathrm{MM}-\mathrm{QM}}\right)r_{\mathrm{MM}-\mathrm{QM}}\; \end{array}$$

where $r_{\mathrm{MM}-\mathrm{QM}}$ is a vector pointing from the QM atom to the MM atom.

### 2.2 Periodic Boundary Conditions

Atoms close to the simulation box edge interact with the atoms on the other side of the box. To avoid double counting of the interactions, no atom in the system should see its periodic copy. This is achieved by ensuring the box size to be bigger than double the longest reaction field cutoff distance. This also applies to ME. In EE, this principle is extended to the whole QM zone, where (a) no QM atom should see a periodic copy of any other QM atom; (b) all QM atoms should see a single periodic copy of the MM atom. A typical violation of this condition is shown in Figure S1. The program verifies it every simulation step and terminates the simulation if a periodic copy contact occurs.

## 3 Implementation Details

GROMOS is fundamentally a MM program, and efficiently interfacing it with QM programs requires careful attention to certain details. In classical simulations, the system topology explicitly includes many‐body interaction terms. However, in QM calculations, interactions are computed ab initio, and the user typically only needs to specify the computational method and provide a complete list of atoms with their atomic numbers. When using a united atom topology, such as aliphatic carbons in GROMOS force fields, the implicit hydrogen atoms must be explicitly defined. Alternatively, all‐atom force fields such as GAFF and OpenFF are also supported by the GROMOS MD engine [32].

The QMMM engine of GROMOS is programmed purely in C++. The QM calculations are offloaded to an external QM program. The code is written such that an implementation of additional QM program interface requires only a basic level of C++ knowledge by using the existing implementations as examples. The xtb and Schnetpack interfaces are linked directly to the GROMOS code. All the other interfaces rely on I/O, where GROMOS takes full control over writing the input files, executing the external program and reading the output files in the user‐specified directory. The directory should ideally be located on a virtual shared memory filesystem that resides in RAM, to avoid significantly slower I/O to the physical drive. Unlike full embedding of external libraries, this approach lets users choose their preferred QM program, fully customize its settings, while the source code remains largely independent of the external programs' versions and development. The I/O through RAM usually takes only a negligible fraction of time with respect to the SCF procedure in the QM program. Especially for small QM zones of up to 20 atoms we found a significant increase in simulation speed over file‐based communication approaches. Additional significant speed‐up is achieved by using the highly efficient xtb program, which is directly embedded in GROMOS via its C API.

If the user does not provide the paths, GROMOS automatically creates uniquely named files in the directory specified in the $TMPDIR environment variable and deletes them at the end of the simulation. GROMOS can also retain the converged coefficients from the QM SCF procedure at the previous timestep and use them as the initial guess for the next timestep. Since the change in positions between timesteps is small, this allows much faster SCF convergence in subsequent steps. In the very first step, the initial guess is usually not available, so GROMOS modifies the input for the first timestep accordingly. GROMOS writes the atom types, positions of the QM atoms to the input files. After the QM program finishes, GROMOS parses its output and retrieves the total energy and forces on all particles.

In case of MECC and MEDC, QM/MM interactions are calculated completely on the GROMOS side. In MEDC also partial charges of the QM atoms can be updated from the QM calculations. This is achieved by using MEDC by setting NTQMMM = 1. Although tempting, we discourage from using this approach for longer simulations, since the fluctuating charges, which are now a function of the positions of the atoms, impose extra forces on the QM atoms. These are not being calculated by default, because depending on the charge assignment scheme, they are often not trivial. Moreover, as discussed in Supporting Information and shown in Figure S2, this term seems to be dominant with respect to the standard Coulombic term. Therefore, using MECC (NTQMMM = −1) or EE (NTQMMM = 2) should be preferred.

In case of EE, also positions and charges of the MM atoms are written to the QM program input. The MM atoms are treated as point charges in the SCF procedure of the QM program. Depending on the QM program, the forces on the MM atoms are provided in three different ways: (1) the forces on the point charges are provided directly (MNDO program, DFTB, ORCA, Turbomole, xtb), or (2) the electric field at the particles positions is provided (Gaussian), and GROMOS then calculates forces of the *i*‐th particle as:

(10) $$\begin{array}{c} f_{i}=q_{i}E_{i}\; \end{array}$$

or (3) charges on the QM atoms are provided (MOPAC) and forces are then calculated for all *M* × *N* QM–MM pairs in GROMOS as:

(11) $$\begin{array}{c} f_{\mathrm{ij}}=\sum_{i}^{M}\sum_{j}^{N}\frac{q_{i}q_{j}}{4\pi \varepsilon_{0}d_{\mathrm{ij}}^{3}}r_{\mathrm{ij}}\; \end{array}$$

In the latter case, GROMOS provides the electric field at every QM atom to MOPAC to include it in the one‐electron Hamiltonian elements [19, 33, 34].

### 3.1 From the Force Field to the QM Atoms

Since QM calculations require the explicit positions of all atoms, the user must provide a list containing the atomic numbers of all atoms, including those from the united atom topology if such a force field is being used. Optionally, bonds to MM atoms can also be included in the same list, with a maximum of single link per QM atom. During the initialization, GROMOS performs several consistency checks. In case a bond across the QM/MM boundary exists, the user must specify the MM atom to which the QM atom is bound. Consistency of all classical bonded terms is checked and if the term is entirely described by the QM calculation, it is removed from the MM topology. The program also simultaneously checks, if all the links between the bonded QM–MM atoms are specified in the QM zone specification. The terms removed from the topology to be treated solely by the QM calculation are summarized in Figure 1A. Essentially all 2‐bonded terms between two QM atoms are removed. For 3‐ and 4‐body terms, we remove all terms where at least the central atoms are in the QM zone. All removed terms are listed in the initialization part of the standard output.

### 3.2 Setting Up a Simulation

Running a QM/MM MD simulation is very similar to a standard MM MD simulation. Beside the standard MD parameters settings, the user specifies the atoms, that should be treated at the QM level, and the parameters for the QM program. The QMMM block of the input parameter file defines the embedding scheme, method, cutoff, frequency of the special trajectory writing, decision to include parametric Lennard‐Jones interactions and/or distance constraints between the QM atoms, and optionally, the scaling of the MM charges in the QM calculation. In a separate file, the qmm file, additional blocks must be specified. In the QMZONE block, the user provides the charge and spin multiplicity of the QM zone, the QM atoms, their atomic numbers, links to the MM atoms. The list of QM atoms has to be provided similar to the cnf file format, where first 17 characters are ignored, and then the atom topology index, atomic number and linked MM atom are specified.

Next, since the QM programs often use different units, their conversion factors for lengths, energies, forces and charges can be respectively specified in the QMUNIT block. The data from QM programs are multiplied and from the GROMOS program divided by them. The block CAPLEN specifies the constant bond length between the capping hydrogen atom and the QM atom in the link atom scheme. The rest of the blocks are QM‐program‐specific. For example, if the MNDO program is specified, the user must provide the MNDOHEADER block, which is a template of the header part of the MNDO input file to which the coordinates and other information are appended. User can also specify optional blocks MNDOBINARY with the path to the MNDO program binary. If omitted, GROMOS expects the “mndo” command parent directory to be in the $PATH environment variable. The input and output files can be also written to paths specified in the MNDOFILES block. If the block is not used, GROMOS writes temporary files to the path specified in the $TMPDIR variable and removes them afterwards. On the other hand, user specified files in the MNDOFILES block are not deleted at the end of the simulation, mainly for debugging purposes. The list of available blocks and possible inputs is listed in Table S2 in the Supporting Information.

Since the QM atoms are read from the topology specification, force fields using united atom topology should be handled with care. The easiest option is to expand the united atoms using the expand_ua program in GROMOS++ [35] on the building blocks, that are supposed to be in the QM zone and then use these to make the topology. The parameters of the added hydrogen atoms then must be modified. Usually, a standard hydrogen IAC number and a charge of 0.0 can be used. Since both the heavy atom and the hydrogen are part of the QM zone, the parameters of the bonded interactions will be ignored, but bonds must still be specified to ensure that the GROMOS program correctly identifies all atoms within a single molecule. In all‐atom force field topologies, this issue is entirely avoided.

## 4 Methods

All QM/MM and MM simulations were performed using the recent version of GROMOS software available online [15]. The starting structures of the test systems were prepared from scratch using Pymol [36]. The simulations were performed in MECC, MEDC, and EE schemes. Force‐field parameters were taken from the GROMOS 54A8 parameter set [37], while the solvent was represented by the SPC water model [38]. In the MECC setup, the QM/MM interaction was calculated the same way as in regular MM. In the MEDC setup, the charges of the QM atoms were updated every step. In EE, a cutoff of 1.4 nm was applied around every QM atom to gather MM point charges for the QM calculation every step. Additionally, the van der Waals interactions of the QM atoms with the MM atoms were calculated with the 54A8 parameters. The QM atoms bond lengths were not constrained, and their mutual Lennard‐Jones interactions were turned off.

The starting structures of the periodic systems, that is, QM water, and amino acids in a rectangular box of SPC water, were prepared using the GROMOS++ program tools [35]. The systems with charged amino acids were neutralized by adding a corresponding number of Na^+^ or Cl^−^ counter ions to the simulation box. We also report results for non‐neutralized boxes. The systems were then minimized and equilibrated for 100 ps, increasing the temperature from 60 to 300 K every 20 ps. At the same time, position restraints of the solute atoms were decreased by factor 10, from an initial value of 2.5 × 10^4^ kJ/mol/nm^2^. The production runs consist of 1 ns MD simulations with 0.5 fs step at 300 K with SHAKE constraints applied only to the solvent. The temperature was maintained by the Nosé–Hoover chains thermostat [39] with relaxation times of 0.1 ps and the solute and solvent degrees of freedom being coupled to different heat baths. The pressure was kept constant at 1 atm using the weak coupling algorithm with a relaxation time of 0.5 ps and an estimated isothermal compressibility of 4.575 × 10^−4^ (kJ/mol/nm^3^)^−1^. The overall translational and rotational degrees of freedom were reset every 1000 steps. Nonbonded interactions were calculated using a triple‐range group based cutoff scheme. Interactions within a short‐range cutoff of 0.8 nm were calculated at every time step using a pair list that was generated every 2.5 fs. Long‐range interactions within a cutoff of 1.4 nm were calculated at pair list generation and kept constant between updates. To account for electrostatic interactions beyond the 1.4 nm cutoff, a generalized reaction field [40] with a relative dielectric permittivity of 61 [41] was used.

For the description of the QM zone, we used semiempirical and DFT methods. From the semiempirical methods, we used AM1 [42], PM3 [43, 44], OM2 [45, 46], and OM3 [47], implemented in the MNDO program [23] and MOPAC [19]. The QM/MM simulations of amino acids in SPC water were performed using the OM3 method in the MNDO program. For sulfur‐containing molecules, we used the OM2 method instead to overcome the missing parameterization. The tripeptide potential energy scans were obtained by energy minimization of the entire system with the constrained C‐C_α_‐C_β_‐C_γ_ torsion angle. In QM/MM and MM minimizations, we applied dihedral‐angle constraints [48, 49]; for the MOPAC [19] and Gaussian16 [21] calculations, the corresponding internal coordinates were kept frozen. The final torsion angles were within 0.01° of the target value. As a reference method, we used the density functional theory (DFT) with the ωB97XD [50] in the 6‐31G(d) basis set [51, 52, 53] as implemented in Gaussian16 [21]. This method is not overly costly and provides reasonably precise total electronic energies, including a version of Grimme's D2 dispersion model. For the sake of comparison, we also performed the calculations fully with the PM3 method and classically in the GROMOS force field.

## 5 Results and Discussion

### 5.1 Test System 1: QM Water in SPC Water

Our first test system, a liquid water, is described very simply in MM terms within the SPC model and its real physical properties are well documented. By describing a single molecule at the QM level, we can inspect the effect of this change on the mutual interactions with other molecules. Firstly, we aim for the stability of the NPT simulation, which can be evaluated by monitoring potential and kinetic energy, temperature, or density. Figure S3 shows the time series of the system quantities. In MECC and EE, the simulations are stable, and all properties converge. With the MEDC, however, the system becomes unstable, which is apparent from the QM energy plot. The enthalpy of vaporization can be estimated directly from the interaction of the solute molecule with the solvent. Its value for SPC water is 43.7 kJ/mol [54]. As shown in the plots, this is well reproducible only for MECC, where the mutual interaction is calculated classically. Except the simulation with dynamic charges, the properties are reliably converging toward their averages and the simulations are stable. In the case of dynamic charges, the missing charge derivatives terms lead to unphysical behavior. Although these properties are still mainly driven by the MM part of the system, the instability coming from the ill‐defined forces considerably disrupts the system.

To further investigate water–water interactions at the QM/MM level, we performed simulations of a water dimer in vacuo under NVE conditions. The simulation results are summarized in Figure S4. Similar to the condensed‐phase simulations, the MEDC simulations exhibit instability, while the MECC, EE and full QM simulations remain stable. Depending on the method and embedding scheme, the dimer binding energy ranges from −15 to −35 kJ/mol. The SPC model tends to overestimate the binding, whereas the QM methods used in this study underestimate it. Among the methods tested, the OM2/EE approach provides the closest estimate to the reference value of −21 kJ/mol [55].

A water molecule is a very simple system, which allows us to scrutinize its geometric properties in detail, as shown in Table 1. All approaches predict reasonable bond lengths and angles. The experimentally measured equilibrium O—H bond length is 0.0990 nm [56]. The averages observed in our QM/MM simulations are in the range from 0.0953 to 0.1029 nm. The most accurate from the tested approaches were EE/AM1 and EE/OM3. The MEDC approaches predict reasonable bond lengths as well, however the unstable simulations lead to high fluctuations, which is obvious from the listed standard deviations. The least satisfactory prediction came from MECC/OM3, which overestimates the bond length by up to 3.9 pm, and from EE/PM3, that underestimates the value by 3.6 pm.

**TABLE 1:** Property averages and standard deviations over a 1 ns simulation of QM water in SPC water.

Embedding/method	O—H bond length/nm	O—H bond stretching modes/cm^−1^	H—O—H angle/°	H—O—H bending mode/cm^−1^
MECC/AM1	0.0981 ± 0.0020	~3350, ~3450	99.9 ± 3.6	1928
MECC/PM3	0.0968 ± 0.0023	~3670, 3850	103.4 ± 4.3	~1790
MECC/OM2	0.1009 ± 0.0024	3018, 3102	98.3 ± 4.3	1791
MECC/OM3	0.1029 ± 0.0027	~3320, 3377	100.3 ± 4.2	~1750
MEDC/AM1	0.0964 ± 0.0007	3494, 3577	103.0 ± 2.4	1898
MEDC/PM3	0.0953 ± 0.0007	3869, 3994	107.1 ± 2.9	1755
MEDC/OM2	0.1001 ± 0.0083	~2980, 3136	101.4 ± 9.2	~1700
MEDC/OM3	0.1020 ± 0.0062	3469, 3519	104 ± 11	1698
EE/AM1	0.0966 ± 0.0012	3469, 3569	103.4 ± 3.2	1875
EE/PM3	0.0954 ± 0.0011	3861, 3994	107.2 ± 3.6	1748
EE/OM2	0.0980 ± 0.0019	3094, 3211	102.5 ± 4.1	1761
EE/OM3	0.1006 ± 0.0025	~3410, 3452	105.0 ± 4.3	~1710
MM/SPC	0.1024 ± 0.0017	3869	104.9 ± 3.0	2111
Exp.	0.0990 (5)^a^	3400^b^	106.6 ± 0.7^c^	

^a^ Reference [56].

^b^ Linewidth of several hundred cm^−1^, reference [57].

^c^ Calculated from intramolecular OD and DD distances reported in reference [58].

Regarding bond angles, no experimental value is available for light water in the liquid state. The closest reference comes from a neutral diffraction experiment on heavy water [58]. To the best of our knowledge, the bond angle of liquid H₂O has not been experimentally measured. However, precise path‐integral simulations for H₂O and D₂O show bond angles of 106.5° and 106.3°, respectively, consistent with the D_2_O experimental value [59]. The EE simulations reproduce accurately these reference values, with EE/OM3 and EE/PM3 being the most accurate. The ME simulations mostly underestimate the angle.

**FIGURE 2:**
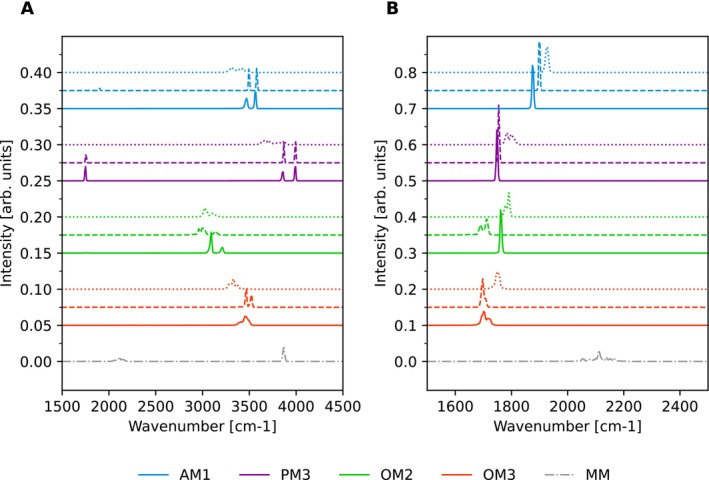
Power spectra of (A) O—H bond stretching vibration and (B) H—O—H bending vibration, obtained from the autocorrelation function of a 1 ns QM/MM simulation. The dotted lines represent mechanical embedding with constant charges (MECC), the dashed lines correspond to mechanical embedding with dynamic charges (MEDC), and the solid lines depicts electrostatic embedding (EE). No additional features were observed outside the plotted range.

In addition to determining equilibrium bond lengths and angles, we analyzed the vibrational dynamics of the QM water, focusing on the O—H bond stretching and H—O—H angle bending modes. The corresponding power spectra were computed as the Fourier transforms of the autocorrelation function of the bond length and bond angle. These results are presented in Figure 2 and summarized in Table 1.

The distinct peak of the O—H bond stretching was observed between 3000 and 4000 cm^−1^, with the lowest prediction from the OM2 method and the highest from the PM3 method. Interestingly, only slight differences could be observed between the MEDC and EE. However, the MECC seems to disrupt the structure of the O—H bond by lowering its frequency and broadening the peak, which can be interpreted as a weakening of the O—H bond. The water molecule possesses two experimentally observable bond stretching modes, the symmetric at 3280 cm^−1^ and antisymmetric at 3410 cm^−1^. The closest to these values were AM1 and OM2. The MM spectrum, calculated from a simulation with a single flexible SPC water molecule in SPC water, shows only a single peak, which is not surprising as the SPC model was parameterized for intermolecular properties with constrained intramolecular structure [38]. This peak covers both modes. The wavenumber of experimental H—O—H angle bending vibration is 1648 cm^−1^. Here most of the methods performed reasonably and predict the peak between 1600 and 2000 cm^−1^. OM2 and OM3 are closest to the experiment. The results are also more consistent among the methods and between different schemes, although the MECC consistently increases the frequency. For the reference, the angle in the SPC water vibrates beyond 2000 cm^−1^ and is the least accurate.

**FIGURE 3:**
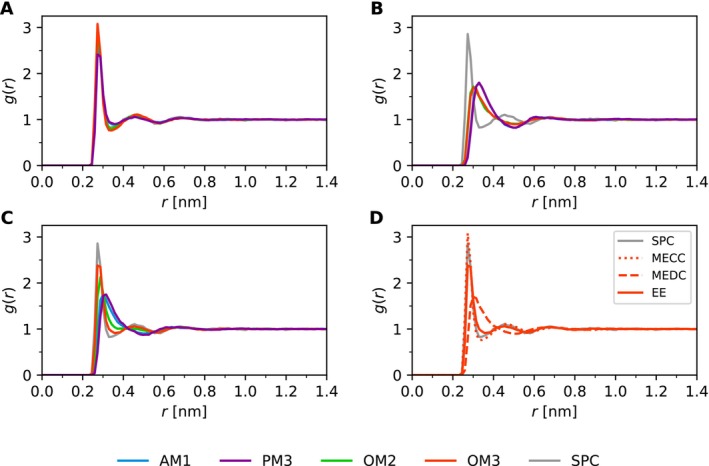
The RDF of 1 ns simulations with (A) mechanical embedding with constant charges (MECC), (B) dynamic charges (MEDC) and (C) electrostatic embedding (EE). Panel (D) shows RDF of OM3 simulation with different embedding schemes. The MM simulation with the SPC water model serves as a reference.

Beside the numerical quantities, the system structure can be well defined and experimentally validated by analysis of the oxygen–oxygen pair radial distribution function (RDF). The RDF plots of the setup combinations are summarized in Figure 3.

The simulated RDF of liquid water exhibits a total of four maxima. The first and second peak are well pronounced, and in classical MD simulations possess maxima at 0.275 nm, and 0.45 nm, respectively [54]. The other two maxima are significantly broadened. This agrees well with the experimental data of liquid water at 300 K, that yield 0.2804 and 0.451 nm, respectively [60]. With no surprise, the mechanical embedding with constant charges (MECC) resembles the MM simulation the most due to the mutual QM/MM interaction being described solely on the MM level. With MEDC, the results are much less structured. The disruption already pronounced in the time series of energies also affects the structure, as shown in the RDF. The electrostatic embedding (EE) simulation gives inconsistent results among different semi‐empirical methods. While the newer OM2 and OM3 are rather consistent with the experiment, RDFs obtained via AM1 and PM3 simulations are distorted similarly to MEDC. The best performing semi‐empirical method in this case is OM3.

**FIGURE 4:**
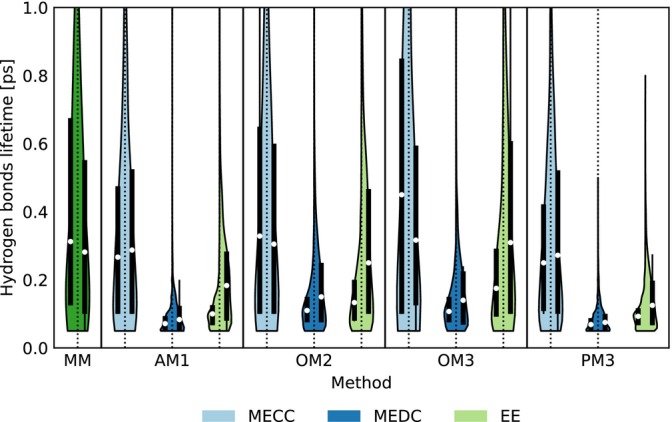
Distributions of hydrogen bond lifetimes between the QM water and SPC water with different embedding schemes and methods. The left‐hand side represents hydrogen bonds where QM water acts as a donor to SPC water, while the right‐hand side shows hydrogen bonds where QM water serves as an acceptor. The white dots indicate the mean value, the black bars represent the interquartile range (2nd and 3rd quartiles), and the whiskers denote adjacent values. The colored areas depict the relative distribution.

The structure of liquid water is primarily governed by hydrogen bonds. Classical force‐field methods have been reported to accurately reproduce this hydrogen‐bonded network [38]. While certain ab initio approaches can reproduce the bonding structure of water molecules [61, 62, 63], simulating liquid water still remains challenging. Water's unique properties, such as hydrogen bonding and its highly flexible molecular structure, often require special considerations or the NVT ensemble to reproduce them under ambient conditions [64, 65, 66]. However, an important question remains: how accurately are these hydrogen bonds reproduced at the QM/MM boundary, where QM and MM methods interface? Under identical settings, the resulting RDF plots, along with other calculated properties such as total electronic energy and hydrogen bond statistics, are effectively identical to those reported in reference [12]. This alignment demonstrates that the new implementation of the code is functionally equivalent. To test the program further, we looked at the lifetime of hydrogen bonds, which tells us more about the dynamics of the QM/MM interactions. Figure 4 displays the lifetime distributions of hydrogen bonds. The average lifetime, as obtained from high‐level ab initio simulations, is 0.78 ps [64]. All tested methods tend to underestimate this lifetime, with OM2 and OM3 in the MECC scheme providing the closest estimates. Both EE and MEDC methods significantly underestimate hydrogen bond lifetimes, with MEDC showing a particularly large discrepancy. Additionally, these embeddings reveal a notable imbalance between donors and acceptors, suggesting a misalignment in the mutual QM/MM interactions.

We also performed simulations of QM water droplets of up to 16 QM molecules in SPC water. As a proof‐of‐concept, we did not apply any restraints, and we kept the initial level of theory of every particle. Most of the simulations resulted in droplet diffusion, making further simulation irrelevant due to the mixing of MM particles within the QM zone. This behavior is desired as no mixing would imply preference of QM and MM water molecules to cluster with their type, thus an imbalance between the levels of theory. Overall, among the semiempirical methods tested, OM3 demonstrates the greatest consistency with reference data under the given conditions.

### 5.2 Test System 2: Amino Acid Residues in SPC Water

A correct description of the interactions between amino acids and water molecules are crucial in correct reproduction of protein dynamics. Including nearby water molecules in the QM region adds high computational cost, therefore we aim for a reasonable description of the interaction between a QM amino acid dissolved in SPC water under NPT conditions.

**FIGURE 5:**
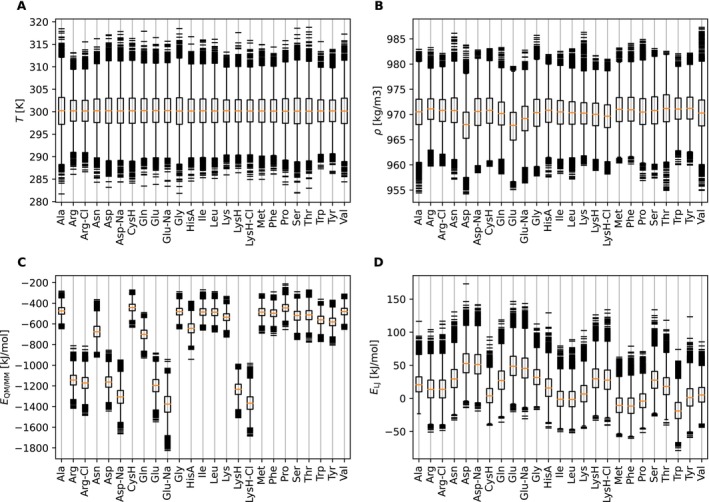
MD Simulations of QM amino acids in SPC water: (A) Box temperature, (B) box density, (C) total QM/MM interaction energy, and (D) total Lennard‐Jones (LJ) interaction energy between solute and solvent.

Firstly, we monitored internal properties of the amino acid molecule in the SPC water environment. The Figure 5 shows QM/MM interaction energy, the Lennard‐Jones contribution, temperature, and density of the simulation box. As shown by the fluctuations of energies in Figure S5, all tested amino acids exhibit stable behavior over the 1 ns simulation period. The fluctuations of the total, potential and kinetic energy are not different from classical MD simulations. The fluctuations of the QM energy are larger for the charged species, nevertheless, their average converges quickly as well. The box temperature is slightly higher than the target, a trend that was also observed in previous simulations using SPC water with a group‐based reaction‐field cutoff and the SHAKE algorithm [67]. To further investigate the noise, we performed a NVE simulation. The observed drift in the energy was the same as in the MM simulation, therefore we conclude that the QM region and QM/MM interface do not induce any significant energy drift. Figure 5 summarizes the monitored observables. The observed box temperatures and densities are stable. The interaction energies converge quickly to the mean and no discrepancies were observed.

**FIGURE 6:**
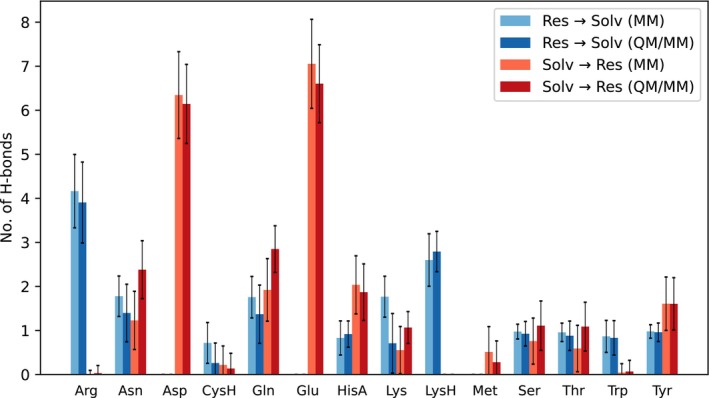
Average number and standard deviation of hydrogen bonds between solute side chains and solvent. All results of the QM/MM are comparable to the MM setup except asparagine, glutamine and lysine. For asparagine and glutamine one more hydrogen bond is accepted on average in the QM/MM simulation. In neutral lysine, one additional hydrogen bond is donated in MM simulation.

Hydrogen bonds are one of the most important stabilizing interactions in proteins [68]. Figure 6 shows hydrogen bonds between the amino acids and water and a comparison between the QM/MM and purely MM approaches. We observed only a subtle difference between the approaches with respect to the number of hydrogen bonds, however, few of the systems behaved differently. In asparagine and glutamine, the average number of hydrogen bonds is nearly one higher, but this difference is insignificant when considering the standard deviation. Compared to the MM simulations, the QM/MM simulations more closely match the theoretical number of hydrogen bonds expected from an ideal hydrogen bond structure.

**FIGURE 7:**
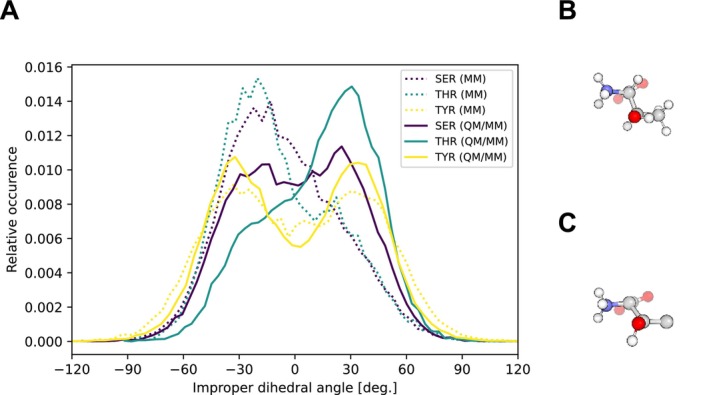
Improper dihedral angles of hydrogen bonds in hydroxylic residues as acceptors. The angles are defined and measured as O—C—H˙˙˙H_b_, where C is the carbon atom adjacent to the hydroxylic oxygen atom, H is the hydroxylic hydrogen atom and H_b_ is the hydrogen atom donated by the SPC water molecule. Panel A shows the relative occurrence of the angles comparing the QM/MM and MM approach. There is a significant discrepancy between the approaches for serine and threonine. Panel B and C show preferred conformation of threonine hydroxyl group in the QM/MM (Panel B) and MM setup (Panel C). The oxygen forms internal hydrogen bond with the amino group, leaving only one position open for the SPC water. Both conformations open and close multiple times during the simulation.

One aspect, where MM simulations may fall behind QM/MM simulations is the preferred position of an accepted hydrogen bond. In a QM description, the donated hydrogen will preferentially occupy the position of the lone pairs on the hydrogen bond acceptor, typically leading to a tetrahedral arrangement around an OH‐group. In a MM description, spherically symmetric potentials are used, and a subtle balance between Coulombic and Lennard‐Jones potentials of the surrounding atoms may lead to a tetrahedral arrangement for steric reasons. In Figure 7A, we analyze the improper dihedral defining the orientation of a donated hydrogen bond. A perfect tetrahedral arrangement around the oxygen, leads to values of this improper dihedral angle of ±35°. While both the MM and the QM/MM descriptions of tyrosine show a largely tetrahedral arrangement, there seem to be (partially opposing) biases in both descriptions for serine and threonine. This can be explained by the dominant conformations observed in both descriptions (exemplified in Figure 7B,C for threonine).

**FIGURE 8:**
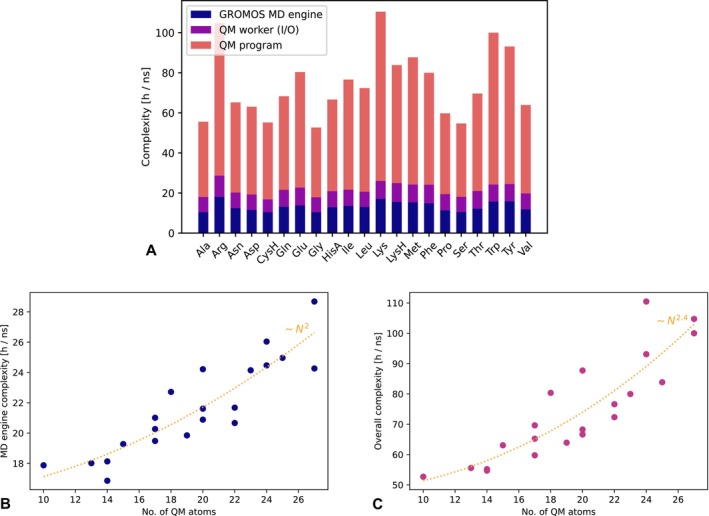
Computational performance evaluation of QM/MM simulation with electrostatic embedding in GROMOS using the OM3 method of the MNDO program shows feasible wall time for a nanosecond simulation. Panel A shows comparison of the simulations of different amino acids. The time accounted to the QM program represents the net wall time of the MNDO program run. The QM worker (I/O) represents the wall time spent writing and reading the input/output files to the virtual filesystem residing in the RAM. The rest, such as MM calculations, MM charges gathering, and others are accounted in the MD engine. Panel B shows computational complexity of the MD engine with the QM worker and I/O, while in panel C the overall complexity is depicted. The spread of points along the y‐axis is primarily due to the heterogeneity of the computer cluster used for the simulations.

We successfully completed every simulation within tens of hours using mid to high‐end hardware configurations, demonstrating the feasibility of achieving a nanosecond time scale. As shown in Figure 8, for a nanosecond simulation of a QM amino acid in SPC water with a 0.5 fs timestep requires between 50 and 110 h/ns. The system sizes range from 10 and 27 QM atoms and 4719 to 7269 MM atoms. This corresponds to 90–200 ms per step, which is a significant improvement against 590 ms per step reported in previous work for a simulation of QM water in SPC water [12]. The speedup, however, primarily comes from the use of a shared memory filesystem and the reuse of coefficients from the previous step. Clearly, the overall performance is primarily limited by the QM program. In these simple simulations, the QM program alone accounted for 60%–95% of the simulation time, making the GROMOS engine's runtime negligible. In practically relevant simulations, the QM zone should be certainly larger, which would lead to even higher fraction of time spent in the QM program. With respect to the number of QM atoms *N*, the measured overall complexity grows with ca. ~*N* ^2.4^ (Figure 8C), whereas the GROMOS MD engine overhead with the QM worker grows only with ~*N* ^2^ (Figure 8B). Note that the number of MM atoms increases with *N*. The use of a naïve double loop pair list algorithm between the QM and MM atoms and the periodic boundary condition violation check accordingly leads to a roughly quadratic scaling. The use of a shared memory filesystem significantly improves I/O performance.

### 5.3 Torsional‐Angle Potential of Amino Acid Residues

To test the implementation of the link atom scheme, we performed the minimal potential energy scan with respect to the torsional angle across the bond across the QM/MM interface. We tested the implementation on tripeptides terminated on both sides by alanine. The central residue is aspartic acid, phenylalanine, histidine, threonine or valine. Below, we will refer to these tripeptides as ADA, AFA, AHA, ATA, and AVA, respectively. Aspartic acid represents a typical polar acidic group. Phenylalanine covers the aromatic and bulky residues, while histidine is a case of bulky and basic residue. Threonine is a small polar residue and finally, valine represents non‐polar, aprotic residues. Since we are interested mainly in the correct description of the torsional angle across the link and the surrounding environment, that is affected by the link scheme the most, we performed minimal potential energy scans of the rotation around the corresponding torsional angle in vacuo. In these tripeptides, the central residue starting from the C_β_ atom is described by the QM method. Both alanines, and the backbone including the C_α_ atom of the central residue are described by the GROMOS 54A8 force field. The interaction between them is described by the electrostatic embedding and the link atom scheme, that is, the classical region is represented by point charges in the QM calculation. Additionally, the QM and MM regions interact mutually through Lennard‐Jones potentials parameterized in the GROMOS 54A8 force field.

**FIGURE 9:**
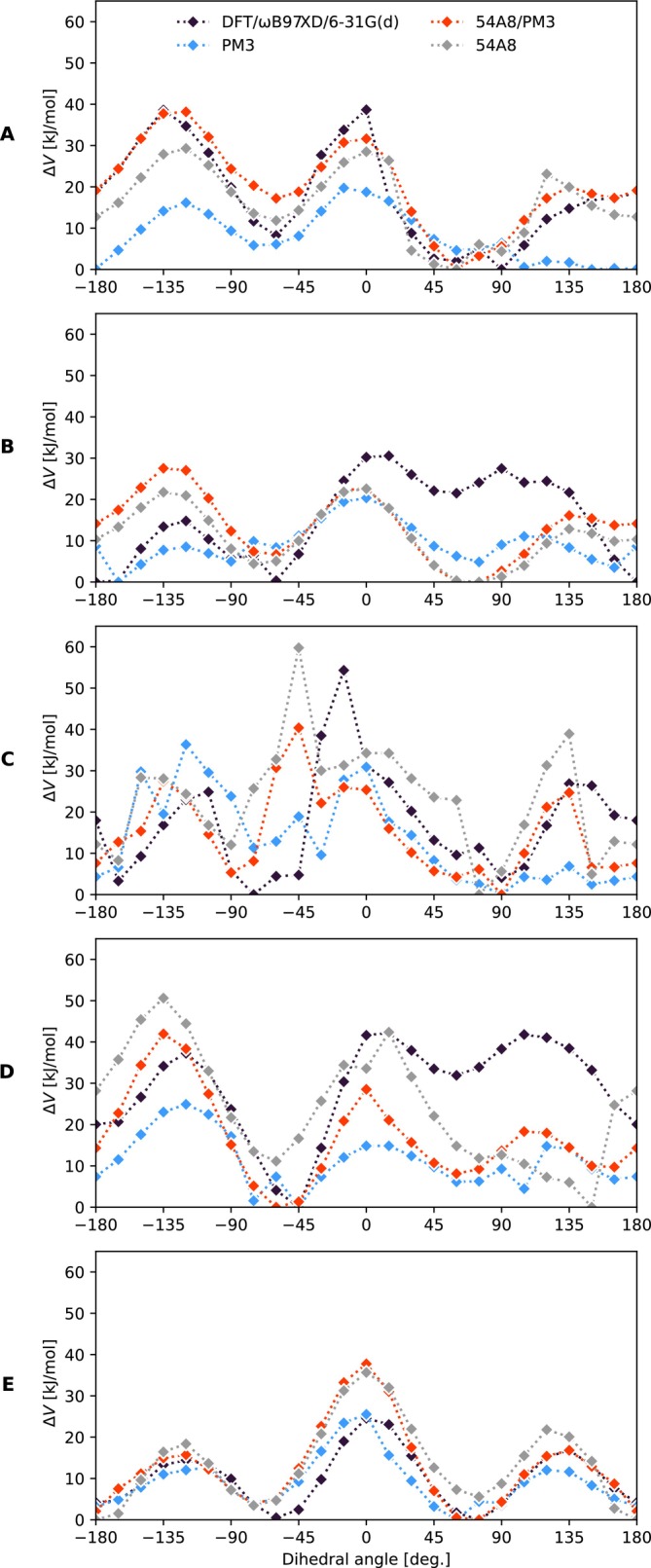
The relative potential energy scans of C‐C_α_‐C_β_‐C_γ_ torsion angle in (A) ADA, (B) AFA, (C) AHA, (D) ATA, and (E) AVA, show reasonable agreement with reference DFT and MM calculations. The noisy region of AHA between −180° and 0° is a result of steric hindrance of the bulky histidine group with the backbone atoms. Depending on the minimization algorithm, different minima were reached. The calculated total potential energies were shifted such that the minimum of every potential energy profile is zero.

The obtained potential energy scans are summarized in Figure 9. Overall, all the methods used reasonably capture the general trends. For ADA, they consistently identify the position of the minimal potential energy between 45° and 90°. They also largely agree on the positions and heights of the energy barriers; however, the PM3 method fails to detect the barrier between 90° and 180°. Interestingly, both the QMMM and MM methods closely follow the curve obtained from the DFT method within this range. In the range of −180° to 0°, the deviation between methods is greater but still acceptable, as the overall shapes of the curves remain similar, with differences primarily in the barrier heights. The best agreement between methods was observed for AVA, which can be easily explained by the absence of significant interactions between the non‐polar aliphatic side chain and the classical backbone. These interactions are driven mainly by van der Waals forces, for which force field methods are generally adequate. The noisy sections, particularly in the AHA profile, result from difficulties in minimizing the bulky structure. The most significant discrepancies in barrier heights were observed for AFA and ATA in the range of 0°–135°, where all methods underestimate the barrier by up to 30 kJ/mol compared to the reference DFT method. This discrepancy could be attributed to the inherent limitations of the lower‐level methods in accurately accounting for the specific electronic interactions within the polar and aromatic side chains present in AFA and ATA. These methods may oversimplify the electron distribution or fail to capture the subtle polarization effects, leading to an underestimation of the energy barriers in comparison to the DFT calculations.

## 6 Conclusions

We presented the improved and extended QM/MM engine of GROMOS, significantly improving its functionality and usability. The updated implementation was extensively tested on several benchmark systems, including QM water in SPC water, amino acids in SPC water, and tripeptides employing the link atom scheme.

The results demonstrate that the new implementation operates as intended. We reported results consistent with the previous implementation, confirming the integrity of the code. Additionally, we provided additional metrics we find important for practical simulations, such as power spectra, hydrogen bond lifetimes, and an in‐depth analysis of program performance. The runtime evaluation shows that the primary computational burden lies within the QM program rather than the QM/MM interface, making the overall lower performance of GROMOS, compared to other MD programs, less significant.

By employing the link atom scheme, the enhanced QM/MM implementation in GROMOS enables more advanced investigations of biomolecular reactivity and enzyme catalysis, which require detailed quantum mechanical treatment within classical MD simulations.

In conclusion, the improvements to the GROMOS QM/MM engine gives more flexibility and control to its users and opens new possibilities for the computational studies in combination with the existing simulation features available in the program.

## Author Contributions

P.P. led the development, including coding, integration and testing of new functionalities, drafted the manuscript and designed the figures. P.B. implemented software interfaces, link atom scheme and designed the user experience workflow. F.P. implemented software interfaces and conducted software testing. S.R. and C.O. provided supervision throughout the development and execution of the project. The manuscript was written through contributions of all authors. All authors have given approval to the final version of the manuscript.

## Conflicts of Interest

The authors declare no conflicts of interest.

## Supporting information


**Data S1.** Supporting Information.

## Data Availability

The data underlying this work, including input files, topologies, coordinates, and scripts used for analysis, are openly available on Zenodo at https://doi.org/10.5281/zenodo.14549977.

## References

[1] W. F. Van Gunsteren , D. Bakowies , R. Baron , et al., “Biomolecular Modeling: Goals, Problems, Perspectives,” Angewandte Chemie‐International Edition 45, no. 25 (2006): 4064–4092. 16761306 10.1002/anie.200502655

[2] W. F. van Gunsteren , X. Daura , P. F. J. Fuchs , et al., “On the Effect of the Various Assumptions and Approximations Used in Molecular Simulations on the Properties of Bio‐Molecular Systems: Overview and Perspective on Issues,” ChemPhysChem 22, no. 3 (2021): 264–282. 33377305 10.1002/cphc.202000968

[3] B. Lier , P. Poliak , P. Marquetand , J. Westermayr , and C. Oostenbrink , “BuRNN: Buffer Region Neural Network Approach for Polarizable‐Embedding Neural Network/Molecular Mechanics Simulations,” Journal of Physical Chemistry Letters 13, no. 17 (2022): 3812–3818. 35467875 10.1021/acs.jpclett.2c00654 PMC9082612

[4] O. T. Unke , S. Chmiela , H. E. Sauceda , et al., “Machine Learning Force Fields,” Chemical Reviews 121, no. 16 (2021): 10142–10186. 33705118 10.1021/acs.chemrev.0c01111 PMC8391964

[5] A. Warshel and M. Levitt , “Theoretical Studies of Enzymic Reactions: Dielectric, Electrostatic and Steric Stabilization of the Carbonium Ion in the Reaction of Lysozyme,” Journal of Molecular Biology 103, no. 2 (1976): 227–249. 985660 10.1016/0022-2836(76)90311-9

[6] M. J. Field , P. A. Bash , and M. Karplus , “A Combined Quantum Mechanical and Molecular Mechanical Potential for Molecular Dynamics Simulations,” Journal of Computational Chemistry 11, no. 6 (1990): 700–733.

[7] D. Bakowies and W. Thiel , “Hybrid Models for Combined Quantum Mechanical and Molecular Mechanical Approaches,” Journal of Physical Chemistry 100, no. 25 (1996): 10580–10594.

[8] H. Lin and D. G. Truhlar , “QM/MM: What Have We Learned, Where Are We, and Where Do We Go From Here?,” Theoretical Chemistry Accounts 117, no. 2 (2007): 185–199.

[9] H. M. Senn and W. Thiel , “QM/MM Methods for Biomolecular Systems,” Angewandte Chemie‐International Edition 48, no. 7 (2009): 1198–1229. 19173328 10.1002/anie.200802019

[10] K. Meier , A. Choutko , J. Dolenc , A. P. Eichenberger , S. Riniker , and W. F. Van Gunsteren , “Multi‐Resolution Simulation of Biomolecular Systems: A Review of Methodological Issues,” Angewandte Chemie‐International Edition 52, no. 10 (2013): 2820–2834. 23417997 10.1002/anie.201205408

[11] U. Ryde , QM/MM Calculations on Proteins, vol. 577, 1st ed. (Methods in Enzymology. Elsevier Inc, 2016), 119–158. 10.1016/bs.mie.2016.05.014 27498637

[12] K. Meier , N. Schmid , and W. F. Van Gunsteren , “Interfacing the GROMOS (Bio)molecular Simulation Software to Quantum‐Chemical Program Packages,” Journal of Computational Chemistry 33, no. 26 (2012): 2108–2117. 22736402 10.1002/jcc.23047

[13] M. Christen , P. H. Hünenberger , D. Bakowies , et al., “The GROMOS Software for Biomolecular Simulation: GROMOS05,” Journal of Computational Chemistry 26, no. 16 (2005): 1719–1751. 16211540 10.1002/jcc.20303

[14] N. Schmid , C. D. Christ , M. Christen , A. P. Eichenberger , and W. F. van Gunsteren , “Architecture, Implementation and Parallelisation of the GROMOS Software for Biomolecular Simulation,” Computer Physics Communications 183, no. 4 (2012): 890–903.

[15] “Biomolecular Simulation – The GROMOS Software [Internet],” https://gromos.net/.

[16] C. Bannwarth , E. Caldeweyher , S. Ehlert , et al., “Extended Tight‐Binding Quantum Chemistry Methods,” WIREs Computational Molecular Science 11, no. 2 (2021): e1493.

[17] K. T. Schütt , P. Kessel , M. Gastegger , K. A. Nicoli , A. Tkatchenko , and K. R. Müller , “SchNetPack: A Deep Learning Toolbox for Atomistic Systems,” Journal of Chemical Theory and Computation 15, no. 1 (2019): 448–455. 30481453 10.1021/acs.jctc.8b00908

[18] W. Jakob , J. Rhinelander , and D. Moldovan , “pybind11 – Seamless Operability Between C++11 and Python,” 2017.

[19] J. J. P. Stewart , “MOPAC2016,” 2016 Colorado Springs, CO, USA: Stewart Computational Chemistry.

[20] B. Hourahine , B. Aradi , V. Blum , et al., “DFTB+, a Software Package for Efficient Approximate Density Functional Theory Based Atomistic Simulations,” Journal of Chemical Physics 152, no. 12 (2020): 124101. 32241125 10.1063/1.5143190

[21] M. J. Frisch , G. W. Trucks , H. B. Schlegel , et al., “Gaussian 16 Revision C.01,” 2016.

[22] F. Neese , “Software Update: The ORCA Program System – Version 5.0,” WIREs Computational Molecular Science 12, no. 5 (2022): e1606.

[23] W. Thiel , “MNDO program. Vol. Version 7. Max‐Planck‐Institut fuer Kohlenforschung,” 2017 Kaiser‐Wilhelm‐Platz 1, Muelheim, Germany; p. Max‐Planck‐Institut für Kohlenforschung: Mülheim a.

[24] S. G. Balasubramani , G. P. Chen , S. Coriani , et al., “TURBOMOLE: Modular Program Suite for Ab Initio Quantum‐Chemical and Condensed‐Matter Simulations,” Journal of Chemical Physics 152, no. 18 (2020): 184107. 32414256 10.1063/5.0004635 PMC7228783

[25] K. E. Shaw , C. J. Woods , and A. J. Mulholland , “Compatibility of Quantum Chemical Methods and Empirical (MM) Water Models in Quantum Mechanics/Molecular Mechanics Liquid Water Simulations,” Journal of Physical Chemistry Letters 1, no. 1 (2010): 219–223.

[26] K. Meier , W. Thiel , and W. F. Van Gunsteren , “On the Effect of a Variation of the Force Field, Spatial Boundary Condition and Size of the QM Region in QM/MM MD Simulations,” Journal of Computational Chemistry 33, no. 4 (2012): 363–378. 22180225 10.1002/jcc.21962

[27] C. M. Clemente , L. Capece , and M. A. Martí , “Best Practices on QM/MM Simulations of Biological Systems,” Journal of Chemical Information and Modeling 63, no. 9 (2023): 2609–2627. 37100031 10.1021/acs.jcim.2c01522

[28] M. Klähn , S. Braun‐Sand , E. Rosta , and A. Warshel , “On Possible Pitfalls in Ab Initio Quantum Mechanics/Molecular Mechanics Minimization Approaches for Studies of Enzymatic Reactions,” Journal of Physical Chemistry B 109, no. 32 (2005): 15645–15650. 16852982 10.1021/jp0521757 PMC1514348

[29] D. Riccardi , P. Schaefer , Y. H. Yang , et al., “Development of Effective Quantum Mechanical/Molecular Mechanical (QM/MM) Methods for Complex Biological Processes,” Journal of Physical Chemistry B 110, no. 13 (2006): 6458–6469. 16570942 10.1021/jp056361o

[30] T. P. Straatsma and J. A. McCammon , “Molecular Dynamics Simulations With Interaction Potentials Including Polarization Development of a Noniterative Method and Application to Water,” Molecular Simulation 5, no. 3–4 (1990): 181–192.

[31] P. H. König , M. Hoffmann , T. Frauenheim , and Q. Cui , “A Critical Evaluation of Different QM/MM Frontier Treatments With SCC‐DFTB as the QM Method,” Journal of Physical Chemistry B 109, no. 18 (2005): 9082–9095. 16852081 10.1021/jp0442347

[32] M. P. Oliveira , Y. M. H. Gonçalves , S. K. Ol Gheta , S. R. Rieder , B. A. C. Horta , and P. H. Hünenberger , “Comparison of the United‐ and All‐Atom Representations of (Halo)alkanes Based on Two Condensed‐Phase Force Fields Optimized Against the Same Experimental Data Set,” Journal of Chemical Theory and Computation 18, no. 11 (2022): 6757–6778. 36190354 10.1021/acs.jctc.2c00524 PMC9648188

[33] V. Luzhkov and A. Warshel , “Microscopic Models for Quantum Mechanical Calculations of Chemical Processes in Solutions: LD/AMPAC and SCAAS/AMPAC Calculations of Solvation Energies,” Journal of Computational Chemistry 13, no. 2 (1992): 199–213.

[34] N. V. Plotnikov and A. Warshel , “Exploring, Refining, and Validating the Paradynamics QM/MM Sampling,” Journal of Physical Chemistry B 116, no. 34 (2012): 10342–10356. 22853800 10.1021/jp304678d PMC12401620

[35] A. P. Eichenberger , J. R. Allison , J. Dolenc , et al., “GROMOS++ Software for the Analysis of Biomolecular Simulation Trajectories,” Journal of Chemical Theory and Computation 7, no. 10 (2011): 3379–3390. 26598168 10.1021/ct2003622

[36] The PyMOL Molecular Graphics System (3.0.0) (Schrödinger, LLC, 2024).

[37] M. M. Reif , P. H. Hünenberger , and C. Oostenbrink , “New Interaction Parameters for Charged Amino Acid Side Chains in the GROMOS Force Field,” Journal of Chemical Theory and Computation 8, no. 10 (2012): 3705–3723. 26593015 10.1021/ct300156h

[38] H. J. C. Berendsen , J. P. M. Postma , W. F. van Gunsteren , and J. Hermans , “Interaction Models for Water in Relation to Protein Hydration,” In Intermolecular forces: proceedings of the fourteenth Jerusalem symposium on quantum chemistry and biochemistry held in jerusalem, israel, april 13–16, 1981, ed. B. Pullman (Netherlands: Springer Netherlands, 1981), 331–342.

[39] G. J. Martyna , M. L. Klein , and M. Tuckerman , “Nosé–Hoover Chains: The Canonical Ensemble via Continuous Dynamics,” Journal of Chemical Physics 97, no. 4 (1992): 2635–2643.

[40] I. G. Tironi , R. Sperb , P. E. Smith , and W. F. van Gunsteren , “A Generalized Reaction Field Method for Molecular Dynamics Simulations,” Journal of Chemical Physics 102, no. 13 (1995): 5451–5459.

[41] T. N. Heinz , W. F. van Gunsteren , and P. H. Hünenberger , “Comparison of Four Methods to Compute the Dielectric Permittivity of Liquids From Molecular Dynamics Simulations,” Journal of Chemical Physics 115, no. 3 (2001): 1125–1136.

[42] M. J. S. Dewar , E. G. Zoebisch , E. F. Healy , and J. J. P. Stewart , “Development and Use of Quantum Mechanical Molecular Models. 76. AM1: A New General Purpose Quantum Mechanical Molecular Model,” Journal of the American Chemical Society 107, no. 13 (1985): 3902–3909.

[43] J. J. P. Stewart , “Optimization of Parameters for Semiempirical Methods I. Method,” Journal of Computational Chemistry 10, no. 2 (1989): 209–220.

[44] J. J. P. Stewart , “Optimization of Parameters for Semiempirical Methods II. Applications,” Journal of Computational Chemistry 10, no. 2 (1989): 221–264.

[45] W. Weber , “Ein neues semiempirisches NDDO‐Verfahren mit Orthogonalisierungskorrekturen: Entwicklung des Modells, Parametrisierung und Anwendungen,” 1996 Universität Zürich, Zürich, Switzerland.

[46] W. Weber and W. Thiel , “Orthogonalization Corrections for Semiempirical Methods,” Theoretical Chemistry Accounts: Theory, Computation, and Modeling (Theoretica Chimica Acta) 103, no. 6 (2000): 495–506.

[47] M. Scholten , “Semiempirische Verfahren mit Orthogonalisierungskorrekturen: Die OM3 Methode,” 2003 rsität Düsseldorf, Düsseldorf, Germany.

[48] M. Christen , A. P. E. Kunz , and W. F. van Gunsteren , “Sampling of Rare Events Using Hidden Restraints,” Journal of Physical Chemistry B 110, no. 16 (2006): 8488–8498. 16623536 10.1021/jp0604948

[49] M. Pechlaner and W. F. van Gunsteren , “Algorithms to Apply Dihedral‐Angle Constraints in Molecular or Stochastic Dynamics Simulations,” Journal of Chemical Physics 152, no. 2 (2020): 024109. 31941329 10.1063/1.5124923

[50] J. D. Chai and M. Head‐Gordon , “Long‐Range Corrected Hybrid Density Functionals With Damped Atom–Atom Dispersion Corrections,” Physical Chemistry Chemical Physics 10, no. 44 (2008): 6615–6620. 18989472 10.1039/b810189b

[51] R. Ditchfield , W. J. Hehre , and J. A. Pople , “Self‐Consistent Molecular‐Orbital Methods. IX. An Extended Gaussian‐Type Basis for Molecular‐Orbital Studies of Organic Molecules,” Journal of Chemical Physics 54 (1971): 724–728.

[52] W. J. Hehre , K. Ditchfield , and J. A. Pople , “Self‐Consistent Molecular Orbital Methods. XII. Further Extensions of Gaussian‐Type Basis Sets for Use in Molecular Orbital Studies of Organic Molecules,” Journal of Chemical Physics 56, no. 5 (1972): 2257–2261.

[53] P. C. Hariharan and J. A. Pople , “The Influence of Polarization Functions on Molecular Orbital Hydrogenation Energies,” Theoretica Chimica Acta 28, no. 3 (1973): 213–222.

[54] A. Glättli , X. Daura , and W. F. van Gunsteren , “Derivation of an Improved Simple Point Charge Model for Liquid Water: SPC/A and SPC/L,” Journal of Chemical Physics 116, no. 22 (2002): 9811–9828.

[55] C. Leforestier , K. Szalewicz , and A. van der Avoird , “Spectra of Water Dimer From a New *Ab Initio* Potential With Flexible Monomers,” Journal of Chemical Physics 137, no. 1 (2012): 014305. 22779646 10.1063/1.4722338

[56] A. Zeidler , P. S. Salmon , H. E. Fischer , J. C. Neuefeind , J. Mike Simonson , and T. E. Markland , “Isotope Effects in Water as Investigated by Neutron Diffraction and Path Integral Molecular Dynamics,” Journal of Physics: Condensed Matter 24, no. 28 (2012): 284126. 22738936 10.1088/0953-8984/24/28/284126

[57] S. T. van der Post , C. S. Hsieh , M. Okuno , et al., “Strong Frequency Dependence of Vibrational Relaxation in Bulk and Surface Water Reveals Sub‐Picosecond Structural Heterogeneity,” Nature Communications 6, no. 1 (2015): 8384. 10.1038/ncomms9384 PMC4595750 26382651

[58] K. Ichikawa , Y. Kameda , T. Yamaguchi , H. Wakita , and M. Misawa , “Neutron‐Diffraction Investigation of the Intramolecular Structure of a Water Molecule in the Liquid Phase at High Temperatures,” Molecular Physics 73, no. 1 (1991): 79–86.

[59] M. Machida , K. Kato , and M. Shiga , “Nuclear Quantum Effects of Light and Heavy Water Studied by All‐Electron First Principles Path Integral Simulations,” Journal of Chemical Physics 148, no. 10 (2018): 102324. 29544339 10.1063/1.5000091

[60] L. B. Skinner , C. J. Benmore , J. C. Neuefeind , and J. B. Parise , “The Structure of Water Around the Compressibility Minimum,” Journal of Chemical Physics 141, no. 21 (2014): 214507. 25481152 10.1063/1.4902412

[61] S. Song , S. Vuckovic , Y. Kim , H. Yu , E. Sim , and K. Burke , “Extending Density Functional Theory With Near Chemical Accuracy Beyond Pure Water,” Nature Communications 14, no. 1 (2023): 799. 10.1038/s41467-023-36094-y PMC9925738 36781855

[62] J. Villard , M. P. Bircher , and U. Rothlisberger , “Structure and Dynamics of Liquid Water From Ab Initio Simulations: Adding Minnesota Density Functionals to Jacob's Ladder,” Chemical Science 15, no. 12 (2024): 4434–4451. 38516095 10.1039/d3sc05828j PMC10952088

[63] A. D. Kaplan , C. Shahi , R. K. Sah , et al., “How Does HF‐DFT Achieve Chemical Accuracy for Water Clusters?,” Journal of Chemical Theory and Computation 20, no. 13 (2024): 5517–5527. 38937987 10.1021/acs.jctc.4c00560

[64] J. Liu , X. He , J. Z. H. Zhang , and L. W. Qi , “Hydrogen‐Bond Structure Dynamics in Bulk Water: Insights From Ab Initio Simulations With Coupled Cluster Theory,” Chemical Science 9, no. 8 (2018): 2065–2073, https://xlink.rsc.org/?DOI=C7SC04205A. 29675248 10.1039/c7sc04205a PMC5885775

[65] L. Ruiz Pestana , N. Mardirossian , M. Head‐Gordon , and T. Head‐Gordon , “Ab Initio Molecular Dynamics Simulations of Liquid Water Using High Quality Meta‐GGA Functionals,” Chemical Science 8, no. 5 (2017): 3554–3565. 30155200 10.1039/c6sc04711d PMC6092720

[66] L. Ruiz Pestana , O. Marsalek , T. E. Markland , and T. Head‐Gordon , “The Quest for Accurate Liquid Water Properties From First Principles,” Journal of Physical Chemistry Letters 9, no. 17 (2018): 5009–5016. 30118601 10.1021/acs.jpclett.8b02400

[67] M. Diem and C. Oostenbrink , “The Effect of Different Cutoff Schemes in Molecular Simulations of Proteins,” Journal of Computational Chemistry 41, no. 32 (2020): 2740–2749. 33026106 10.1002/jcc.26426 PMC7756334

[68] D. Herschlag and M. M. Pinney , “Hydrogen Bonds: Simple after All?,” Biochemistry 57, no. 24 (2018): 3338–3352. 29678112 10.1021/acs.biochem.8b00217

