# Supplementary material for: A Robust and Versatile QM/MM Interface for Molecular Dynamics in GROMOS
Source: J Comput Chem. 2025 Feb 7;46(5):e70053. doi: 10.1002/jcc.70053 (PMC11804165; doi:10.1002/jcc.70053)
Supplement: Supplementary file 1 — Data S1. Supporting Information. [file JCC-46-0-s001.pdf]

Supporting Information for:

A Robust and Versatile QM/MM Interface for Molecular Dynamics in GROMOS

Peter Poliak,[*a,b] *Patrick Bleiziffer,*[c,d] *Felix Pultar,*[c] *Sereina Riniker,*[c] *and Chris Oostenbrink*[a,e]

a. Institute of Molecular Modeling and Simulation, Department of Material Sciences and Process Engineering, University of Natural Resources and Life Sciences, Vienna, Muthgasse 18, Vienna, 1190, Austria

b. Institute of Physical Chemistry and Chemical Physics, Faculty of Chemical and Food Technology, Slovak University of Technology, Radlinského 9, Bratislava, 812 37, Slovakia

c. Department of Chemistry and Applied Biosciences, ETH Zürich, Vladimir-Prelog-Weg 2, 8093 Zürich, Switzerland

d. maXerial AG, Schaanerstrasse 27, 9490 Vaduz, Liechtenstein

e. Christian Doppler Laboratory for Molecular Informatics in the Biosciences, University of Natural Resources and Life Sciences, Vienna, Austria

* Corresponding author: peter.poliak@boku.ac.at

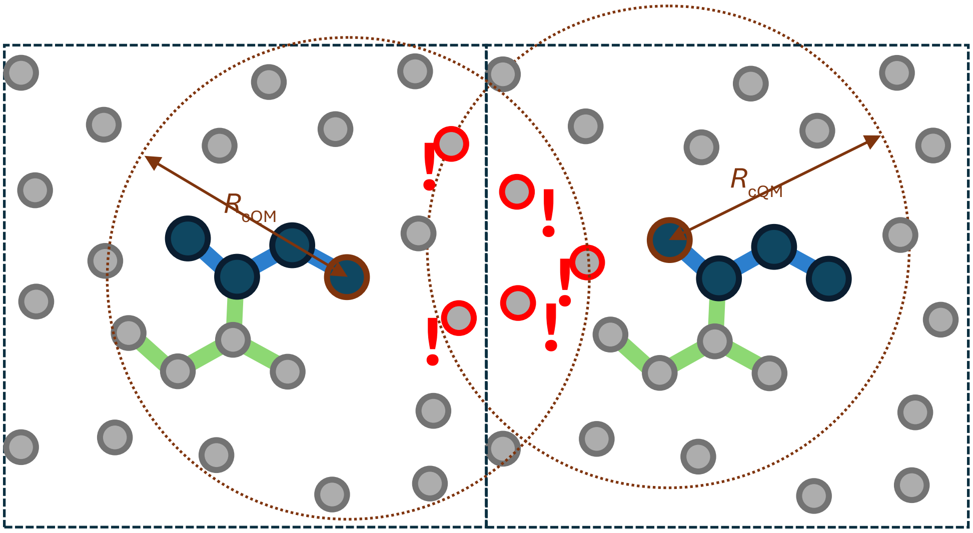

Figure S1. A scheme of periodic boundary conditions (PBC) under QM/MM settings. The QM cutoff distance R_{cQM} is specified by the user. The maximal cut-off rule is extended to all QM atoms, such that all QM atoms should see the same copy of all other QM and MM atoms within the cutoff distance. If this condition is violated, the program terminates.

S1 Mechanical embedding

In mechanical embedding, the QM/MM interaction is treated on the MM level with specific charges assigned to the QM atoms. The QM charges can be taken from the user defined force field parameterization and kept constant during the simulation. As a result, the QM zone is coupled to the MM zone only through the MM-like forces and no mutual polarization takes place. To improve the description, one may update the charges during the simulation, since the QM charge assignment adds only a tiny computational overhead to the SCF procedure. This way a part of the polarization induced by the QM zone conformational change is included. However, the fluctuating charges of the QM zone induce additional forces on the QM atoms from the MM atoms. Moreover, the additional force is not pairwise and depends also on the other polarizable QM atoms. In other words, the Coulombic potential energy term between M MM and N QM atoms is

$$V = \sum_{i}^{M}\sum_{j}^{N}\frac{q_i q_j}{4\pi\varepsilon_0 d_{ij}} \tag{1}$$

By taking the negative gradient to get forces on the atoms, we get

$$-\frac{dV}{d\boldsymbol{r}} = \sum_{i}^{M}\sum_{j}^{N}\frac{q_i q_j}{4\pi\varepsilon_0 d_{ij}^3}\boldsymbol{r}_{ij} - \sum_{i}^{M} q_i \sum_{j}^{N}\frac{1}{d_{ij}}\sum_{k}^{N}\frac{\partial q_j}{\partial \boldsymbol{r}_k} \quad (2)$$

Where *k* runs over all QM atoms. The first term is static, corresponds to typical force field Coulombic term, and depends only on the instantaneous charge values and atomic positions. The second term is dynamic and depends on the polarizabilities of the atoms in the QM zone.

The forces therefore depend on the derivative of the charge with respect to atomic coordinates of all the QM atoms, and their exact values depend on the charge assignment method. The only methods with directly available analytical charge gradients, are Mulliken [1,2], and Löwdin population analysis [3], which are unsuitable due to their heavy dependence on the basis set size. Most practical charge assignment schemes are based on fitting the atomic charges to the molecular electrostatic potential. Since this is an iterative process, analytic charge gradients are often difficult to achieve, and require at least a modification to the charge fitting algorithm, as shown for the CHELPG charges [4]. Numerical derivative evaluation, on the other hand, comes with impractically high computational cost.

We have tested the significance of the second force term of Eq. 2 in a QM/MM simulation of $Fe[H2O]_6^{3+}$. We evaluated the charges using the Merz-Kollmann scheme [5], and trained a Schnet neural network [6] to reproduce charges [7]. The mathematical form of the used neural network allows to obtain the charge derivatives as analytical gradients of the network itself. We found the dynamic term dominant in most of the MM zone, *i.e.* only MM atoms very close to the QM atoms are driven mainly by the static coulombic term. Moreover, the dynamic term is always expected to become significant at a certain distance, since it scales with $\sim 1/r$, whereas the static term scales with $1/r^2$. The ratio between the static and dynamic term depicted in the cross-section of the $Fe[H2O]_6^{3+}$ molecule as a force per unit charge, *i.e.* electric field, is depicted in Fig. 2S.

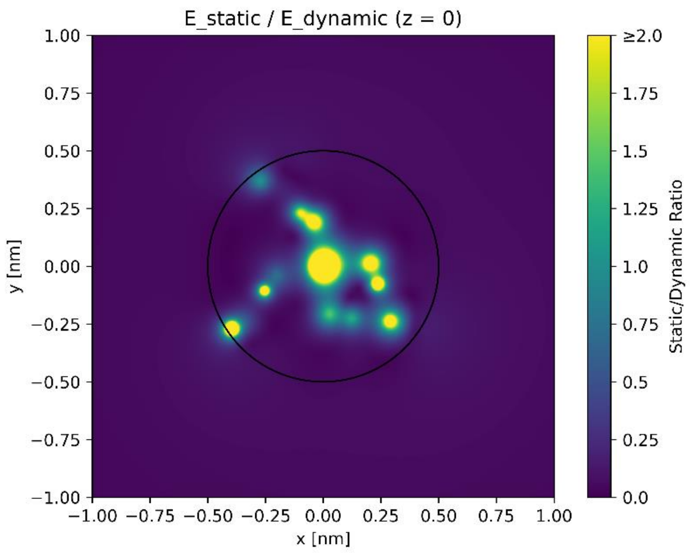

Figure S2. The force caused by QM zone atomic charge changes is dominant against the constant charge coulombic term for most of the MM zone. A cross-section of the $Fe[H2O]_6^{3+}$ molecule, centered on the Fe atom at z=0. The black circle has a radius of 0.5 nm.

Table S1. Compatible versions of QM programs tested with GROMOS. The list is not exhaustive and other versions are compatible if they accept same input and produce same output format.

QM software	Compatible versions
MNDO	7.1
Turbomole	6.3
DFTB+	17.1
	19.1
MOPAC	2016.19.317L
	2016.22.067L
Gaussian 16	Revision B.01
Schnetpack	1.0.0
Orca	4.0.3
	5.0.1
XTB	6.5

Table S2. GROMOS input blocks of the QMMM file specifying the QM calculation details with basic examples. Certain blocks support variables (formatted as `@@VAR@@`), which are dynamically replaced with the corresponding value at each step.

Block name	Description	Content example
`MNDOBINARY`	Command or path to the MNDO program binary.<br>If not specified, the `mndo` command from `$PATH` is used.	`/path/to/mndo`
`MNDOFILES`	Input and output files to exchange data with MNDO (4 lines)<br>If not specified, temporary files are created in `$TMPDIR`. User-specified files are not deleted after use.	`/path/to/mndo.in`<br>`/path/to/mndo.out`<br>`/path/to/mndo_gradient.out`<br>`/path/to/mndo_density.bin`
`MNDOHEADER`	The header part of the MNDO input file.<br>Variables are allowed:<br>• `CHARGE`: net charge of the QM zone<br>• `SPINM`: spin multiplicity of the QM zone<br>• `NUM_CHARGES`: the number of MM atoms<br>• `NUM_LINK`: the number of link and capping atoms	`MNDOHEADER`<br>`kharge=@@CHARGE@@ imult=@@SPINM@@ +`<br>`iop=-8 +`<br>`kitscf=2000 +`<br>`idiis=1 +`<br>`ktrial=11 +`<br>`igeom=1 iform=1 nsav15=4 ipubo=1 jop=-2 +`<br>`mminp=2 mmcoup=2 mmlink=1 nlink=@@NUM_LINK@@`<br>`numatm=@@NUM_CHARGES@@`<br>`title line`
`TMOLEFILES`	Paths, input and output files to run and exchange data with Turbomole (8 lines)	`/path/to/turbomole/binary/directory`<br>`/path/to/working/directory/containing/control/file`<br>`coordinate.in`<br>`mm_coordinate.in`<br>`energy.out`<br>`gradient.out`<br>`mm_gradient.out`<br>`ridft.out`
`TMOLETOOLCHAIN`	Turbomole programs to execute, one line per program	`ridft`<br>`rdgrad`
`DFTBFILES`	Paths, input and output files to run and exchange data with DFTB+ (5 lines)	`/path/to/dftb/binary/directory`<br>`/path/to/working/directory`<br>`coordinate.in`<br>`mm_coordinate.in`<br>`stdout.out`
`DFTBINPUT`	Complete content of the `dftb_in.hsd`	`Geometry = genFormat {`<br>` <<< "coordinate.in"   ## Always required`

	User may provide additional DFTB+ blocks	}<br>Hamiltonian = DFTB {<br><br>## For electrostatic and polarisable embedding also use this block:<br>ElectricField = {<br>PointCharges = {<br>CoordsAndCharges = {<br><<< "mm_coordinate.in"<br>}<br>}<br>}<br>}<br>Analysis = {<br>## ... optional custom settings ...<br><br>CalculateForces = Yes ## Always required<br><br>## For mechanical embedding with dynamic charges, also specify:<br>MullikenAnalysis = Yes<br>}
MOPACBINARY	Command or path to the MOPAC program binary.<br>If not specified, mopac command from $PATH is used.	/path/to/mopac
MOPACFILES	input and output files to exchange data with MOPAC (7 lines)<br>If not specified, temporary files are created in $TMPDIR. User-specified files are not deleted after use.	/path/to/mopac.mop<br>/path/to/mopac.out<br>/path/to/mopac.aux<br>/path/to/mopac.arc<br>/path/to/stdout.out<br>/path/to/mopac.den<br>/path/to/mol.in
MOPACHEADER	the header part of the MOPAC input file.<br>Variables are allowed:<br>• CHARGE: net charge of the QM zone<br>• OLDENS: generates density file in every step using the DENOUT keyword and then reuses it using the OLDENS keyword in subsequent step	PM7 1SCF CHARGE=@@CHARGE@@ GRAD QMMM AUX(PRECISION=9) PRECISE @@OLDENS@@<br>title line
MOPACLINKATOM	Mode of link-atom treatment:<br>• 0 (none) : link atoms see no MM atom (default)	0

	• 1 (exclude_atom) : Link atoms see all MM atoms except the MM atoms involved in the link<br>• 2 (exclude_chargegroup) : Link atoms see all MM atoms except the MM chargegroup involved in the link<br>• 3 (all) : Link atoms see all MM atoms	
GAUBINARY	Command or path to the Gaussian 16 program binary.<br>If not specified, `g16` command from `$PATH` is used.	/path/to/gaussian/binary/g16
GAUFILES	Input and output files to exchange data with Gaussian (2 lines)	/path/to/input.gjf<br>/path/to/output.out
GAUHEADER	The header part of the Gaussian input file	%nproc=8<br>%mem=2GB<br>%NoSave<br>%chk=tmp
GAUROUTE	The route section of the Gaussian input file. The hash (#) is automatically added at the start of the line. Allowed variable:<br>• GUESS: replaced by 'guess=read' after the first step	N hf/STO-3G @@GUESS@@ nosymm pop(mk) force charge(angstroms) prop=(field,read)
GAUCHSM	The net charge and the spin multiplicity of the system. Allowed variables:<br>• CHARGE: net charge of the QM zone calculated automatically<br>• SPINM: spin multiplicity of the QM zone calculated automatically	@@CHARGE@@ @@SPINM@@
NNMODEL	Path to the Schetpack neural network model	/path/to/nn/model
NNVALID	Path to the Schetpack neural network model for the validation	/path/to/nn/validation_model
NNCHARGE	Path to the special charge-predicting Schetpack neural network model	/path/to/nn/charge_model
NNDEVICE	Device to run model on. Possible values:<br>• auto : try CUDA GPU, otherwise use CPU<br>• cpu : use CPU<br>• cuda : use CUDA GPU	cuda
ORCABINARY	Command or path to the ORCA program binary.<br>If not specified, `orca` command from	/path/to/orca/binary/orca

	$PATH is used.	
ORCAFILES	Input and output files to exchange data with ORCA (6 lines)	/path/to/input.inp<br>/path/to/output.out<br>/path/to/coordinates.xyz<br>/path/to/pointcharges.pc<br>/path/to/output_gradient_file.engrad<br>/path/to/output_mm_gradient_file.pcgrad
ORCAHEADER	The header part of the Orca input file.<br>Allowed variables:<br>• CHARGE: net charge of the QM zone calculated automatically<br>• SPINM: spin multiplicity of the QM zone calculated automatically<br>• POINTCHARGES: path to the file containing the point charges (MM atoms)<br>• COORDINATES: File containing the coordinates	! PM3 EnGrad TightSCF<br>%scf MaxIter 1500 end<br>%pointcharges "@@POINTCHARGES@@"<br>* xyzfile @@CHARGE@@ @@SPINM@@ @@COORDINATES@@
XTBOPTIONS		# GFNHAM XVERBO<br>1 1<br># XTBITE XTBACC<br>100 0.1
XTBFILES	(optional) Path to log file	/path/to/xtb.log

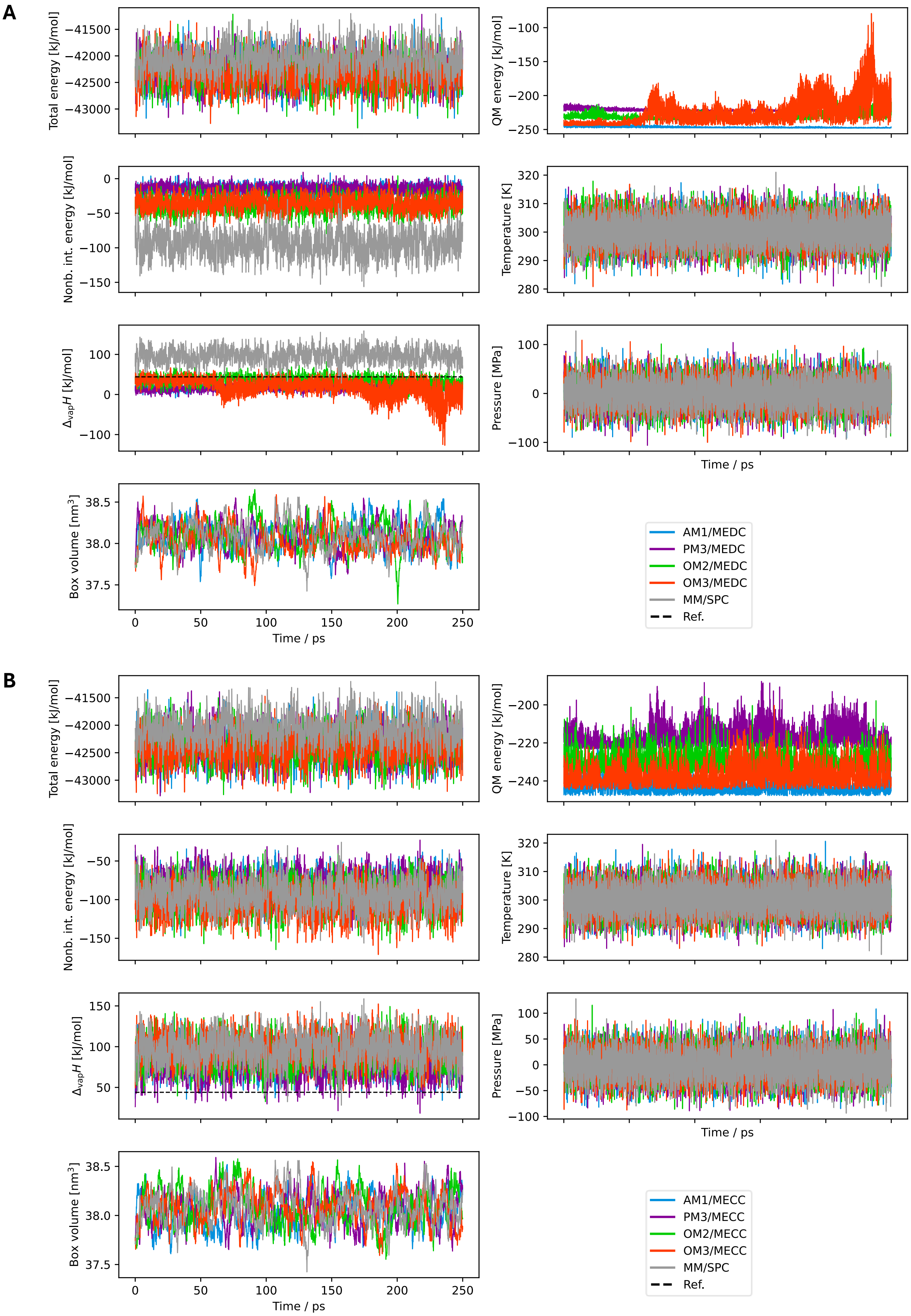
A
Total energy [kJ/mol]
QM energy [kJ/mol]
Nonb. int. energy [kJ/mol]
Temperature [K]
$\Delta_{vap}H$ [kJ/mol]
Pressure [MPa]
Box volume [nm^3]
Time / ps
AM1/MEDC
PM3/MEDC
OM2/MEDC
OM3/MEDC
MM/SPC
Ref.
B
Total energy [kJ/mol]
QM energy [kJ/mol]
Nonb. int. energy [kJ/mol]
Temperature [K]
$\Delta_{vap}H$ [kJ/mol]
Pressure [MPa]
Box volume [nm^3]
Time / ps
AM1/MECC
PM3/MECC
OM2/MECC
OM3/MECC
MM/SPC
Ref.

C

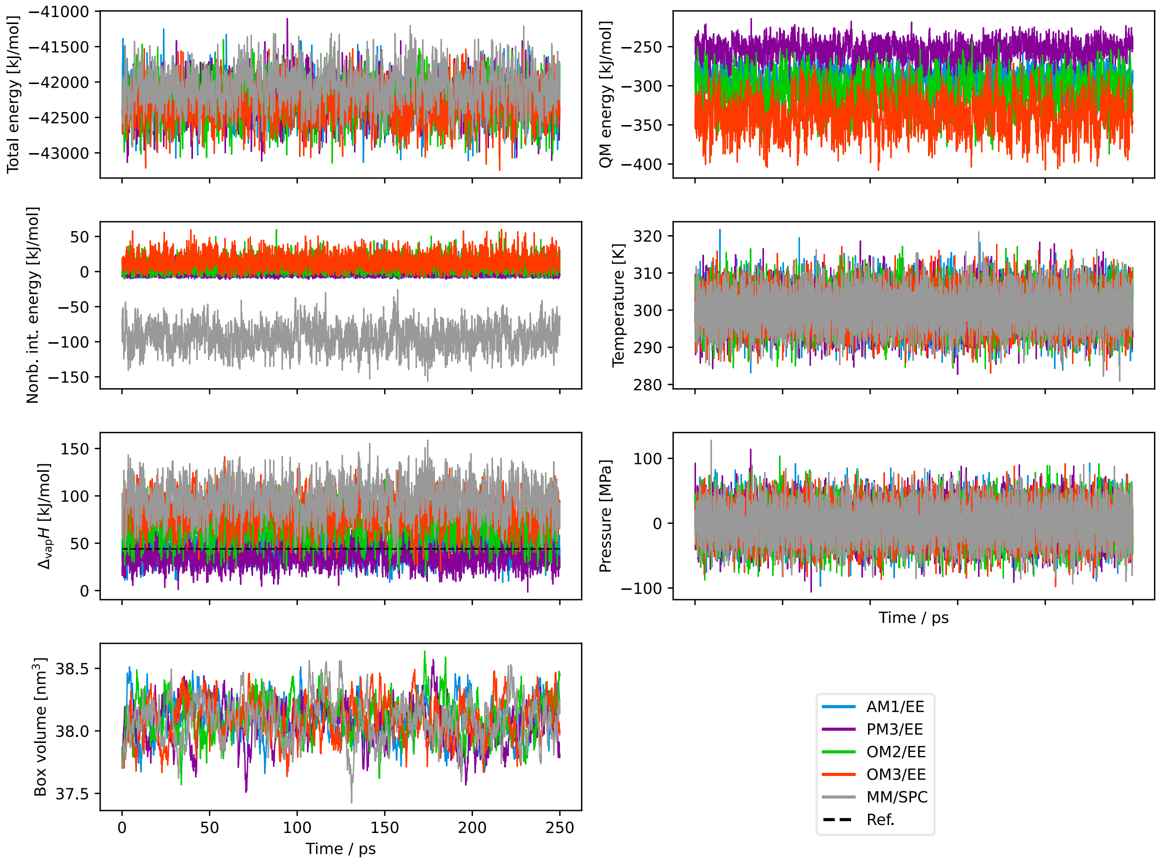

Figure S3. QM/MM MD simulations time series of QM water in SPC water in NPT ensemble. Total energy, QM energy, MM nonbonded interaction energy, temperature, heat of vaporization, pressure and the box volume from the QM/MM simulation with A) mechanical embedding with dynamic charges; B) with constant charges; C) electrostatic embedding. The reference value of the heat of vaporization was taken from ref. [8].

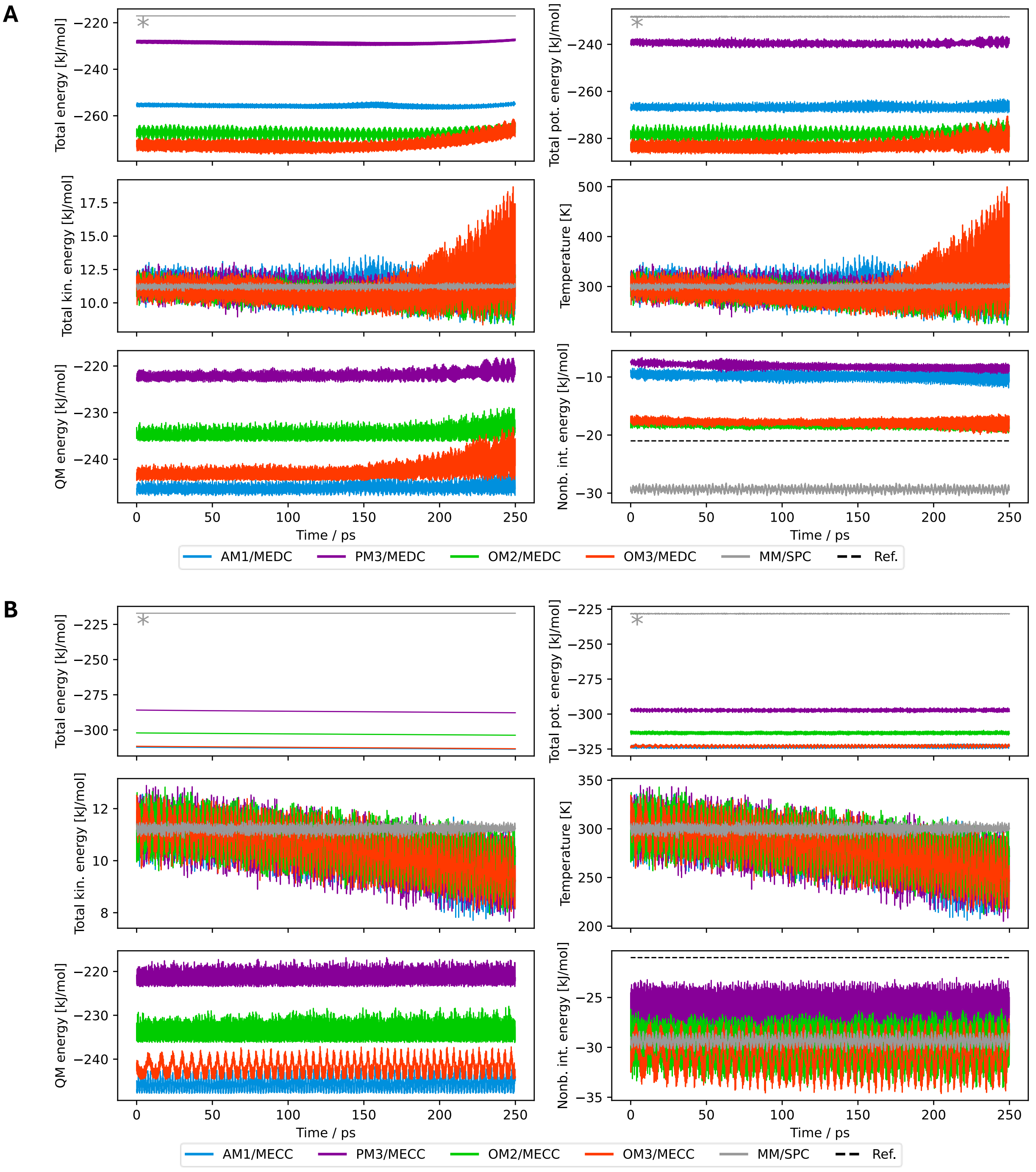
A
Total energy [kJ/mol]
Total pot. energy [kJ/mol]
Total kin. energy [kJ/mol]
Temperature [K]
QM energy [kJ/mol]
Nonb. int. energy [kJ/mol]
Time / ps
AM1/MEDC
PM3/MEDC
OM2/MEDC
OM3/MEDC
MM/SPC
Ref.
B
Total energy [kJ/mol]
Total pot. energy [kJ/mol]
Total kin. energy [kJ/mol]
Temperature [K]
QM energy [kJ/mol]
Nonb. int. energy [kJ/mol]
Time / ps
AM1/MECC
PM3/MECC
OM2/MECC
OM3/MECC
MM/SPC
Ref.

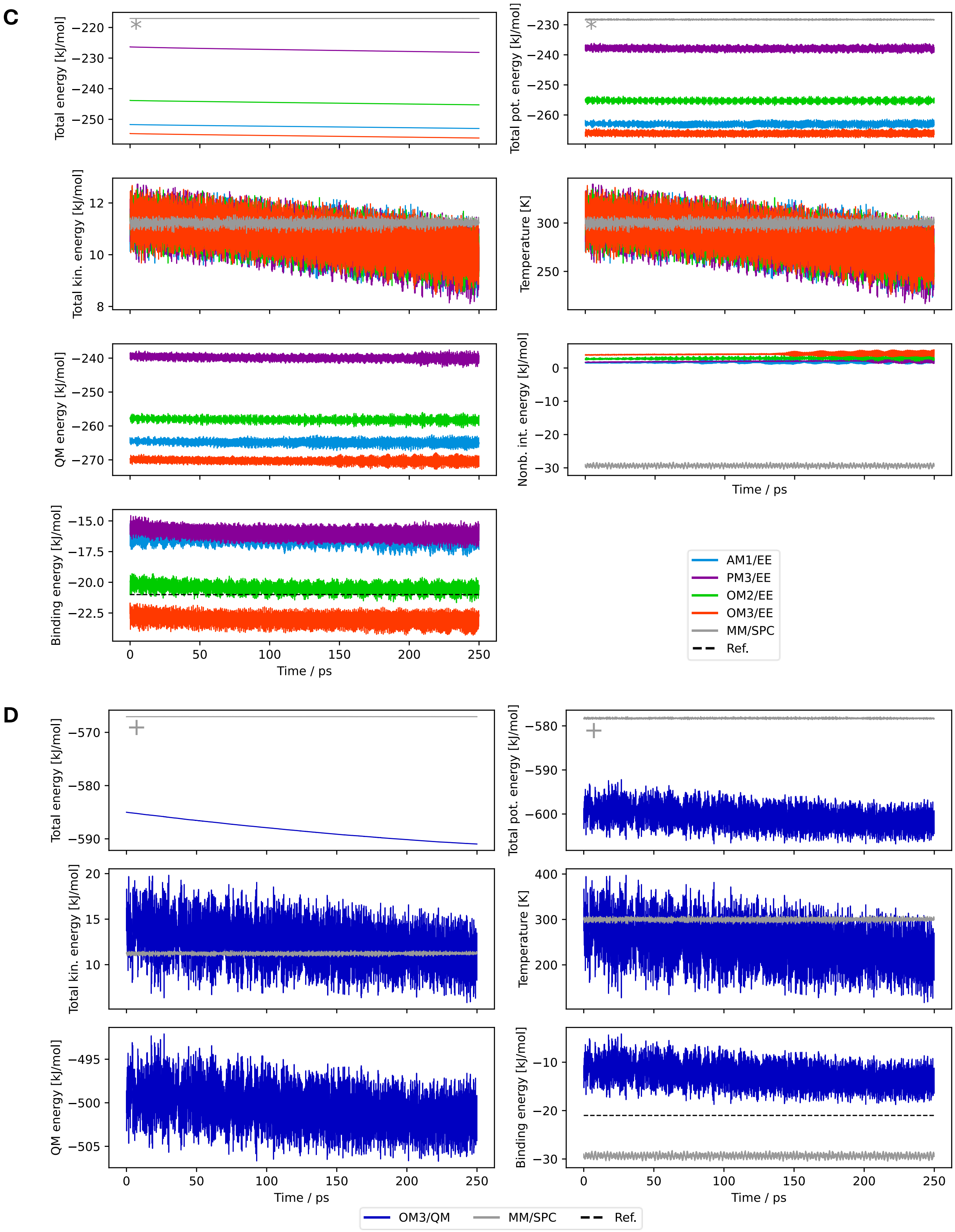

Figure S4. QM/MM MD simulations time series of QM water – SPC water dimer in NVE ensemble *in vacuo*, using a time step of 0.1 fs. Total energy, total potential energy, total kinetic energy, temperature, QM energy, nonbonded interaction energy, and the dimer binding energy, from the QM/MM simulation with A) mechanical embedding with dynamic charges (MEDC); B) with constant charges (MECC); C) electrostatic embedding (EE); D) full QM OM3 simulation. Panel A

shows, that dynamically updated charges of the QM atoms lead to unstable simulations. The observed energy drift in Panels B and C is below 0.008 kJ/mol/ps, and is within the values reported for other QM/MM implementations [9]. The marked lines were shifted by a constant vertical offset of –200 kJ/mol (*) and –550 kJ/mol (+) to facilitate visual comparison. The nonbonded interaction energy includes only MM terms, which in Panels A and B corresponds to the binding energy. In Panel C, we report the binding energy separately, since in EE the QM-MM coulombic interactions are treated on the QM level and the QM-MM dispersion is treated classically using Lennard-Jones potentials. The reference *ab initio* binding energy was taken from ref. [10]. The single water total potential energies used for the calculation of binding energies were obtained as time averages of short vacuum QM MD simulations.

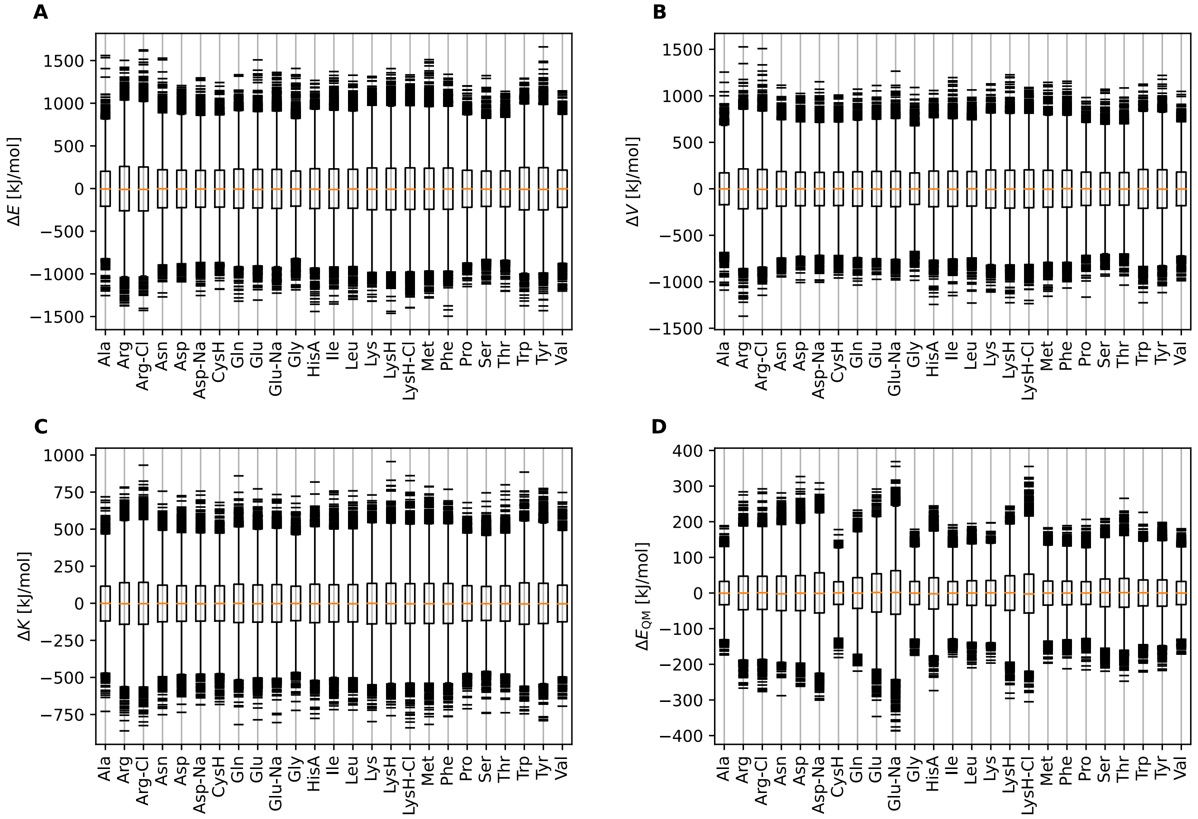

Figure S5. MD Simulations of QM amino acids in SPC water: Fluctuations of (A) total energy, (B) total potential energy, (C) total kinetic energy, and (D) total QM energy.

References

1. Mulliken RS. Electronic Population Analysis on LCAO–MO Molecular Wave Functions. I. J Chem Phys. 1955 Oct 1;23(10):1833–40.

2. Hratchian HP, Parandekar P V., Raghavachari K, Frisch MJ, Vreven T. QM:QM electronic embedding using Mulliken atomic charges: Energies and analytic gradients in an ONIOM framework. J Chem Phys. 2008 Jan 21;128(3).

3. Löwdin PO. On the Non-Orthogonality Problem Connected with the Use of Atomic Wave Functions in the Theory of Molecules and Crystals. J Chem Phys. 1950 Mar 1;18(3):365–75.

4. Herbert JM, Jacobson LD, Un Lao K, Rohrdanz MA. Rapid computation of intermolecular interactions in molecular and ionic clusters: Self-consistent polarization plus symmetry-adapted perturbation theory. Physical Chemistry Chemical Physics. 2012 Jun 7;14(21):7679–99.

5. Besler BH, Merz KM, Kollman PA. Atomic charges derived from semiempirical methods. J Comput Chem. 1990 May;11(4):431–9.

6. Schütt KT, Kessel P, Gastegger M, Nicoli KA, Tkatchenko A, Müller KR. SchNetPack: A Deep Learning Toolbox for Atomistic Systems. J Chem Theory Comput. 2019;15(1):448–55.

7. Lier B, Poliak P, Marquetand P, Westermayr J, Oostenbrink C. BuRNN: Buffer Region Neural Network Approach for Polarizable-Embedding Neural Network/Molecular Mechanics Simulations. Journal of Physical Chemistry Letters. 2022 May 5;13(17):3812–8.

8. Chase MW, Jr, editor. NIST-JANAF thermochemical tables, monograph 9. 4th ed. Vol. 9. Washington DC: American Chemical Society; 1998. 1–1951 p.

9. Melo MCR, Bernardi RC, Rudack T, Scheurer M, Riplinger C, Phillips JC, et al. NAMD goes quantum: An integrative suite for hybrid simulations. Nat Methods. 2018;15(5):351–4.

10. Leforestier C, Szalewicz K, van der Avoird A. Spectra of water dimer from a new *ab initio* potential with flexible monomers. J Chem Phys. 2012 Jul 7;137(1).
